# Bioinformatics methods for identification of amyloidogenic peptides show robustness to misannotated training data

**DOI:** 10.1038/s41598-021-86530-6

**Published:** 2021-04-26

**Authors:** Natalia Szulc, Michał Burdukiewicz, Marlena Gąsior-Głogowska, Jakub W. Wojciechowski, Jarosław Chilimoniuk, Paweł Mackiewicz, Tomas Šneideris, Vytautas Smirnovas, Malgorzata Kotulska

**Affiliations:** 1 Department of Biomedical Engineering, Wroclaw University of Science and Technology, 50-370 Wroclaw, Poland; 2 University of Lorraine, CNRS, 5400 Nancy, France; 3 Medical University of Bialystok, 15-089 Białystok, Poland; 4 Institute of Biochemistry and Biophysics, Polish Academy Sciences, 02-106 Warsaw, Poland; 5 Faculty of Biotechnology, University of Wroclaw, 50-137 Wroclaw, Poland; 6 Life Sciences Center, Institute of Biotechnology, Vilnius University, 01513 Vilnius, Lithuania

**Keywords:** Biophysics, Computational biology and bioinformatics, Molecular biology

## Abstract

Several disorders are related to amyloid aggregation of proteins, for example Alzheimer’s or Parkinson’s diseases. Amyloid proteins form fibrils of aggregated beta structures. This is preceded by formation of oligomers—the most cytotoxic species. Determining amyloidogenicity is tedious and costly. The most reliable identification of amyloids is obtained with high resolution microscopies, such as electron microscopy or atomic force microscopy (AFM). More frequently, less expensive and faster methods are used, especially infrared (IR) spectroscopy or Thioflavin T staining. Different experimental methods are not always concurrent, especially when amyloid peptides do not readily form fibrils but oligomers. This may lead to peptide misclassification and mislabeling. Several bioinformatics methods have been proposed for *in-silico* identification of amyloids, many of them based on machine learning. The effectiveness of these methods heavily depends on accurate annotation of the reference training data obtained from *in-vitro* experiments. We study how robust are bioinformatics methods to weak supervision, encountering imperfect training data. AmyloGram and three other amyloid predictors were applied. The results proved that a certain degree of misannotation in the reference data can be eliminated by the bioinformatics tools, even if they belonged to their training set. The computational results are supported by new experiments with IR and AFM methods.

## Introduction

Amyloids are a group of proteins folding into assemblies of insoluble fibrils of very regular and tightly packed β-structures, which resemble a steric zipper. Despite the importance of amyloids, which is related to their roles in various diseases, their formation and unique behavior are not fully explained^1^. One of the challenges associated with amyloid studies is to establish computationally, whether a protein can form amyloids. Currently available tools addressing this question use statistical and physical models^2,3^. The statistical methods are only based on the amino acid composition of previously annotated amyloid and non-amyloid proteins and use computational models recognizing regularities in the sequences^4–6^. The physical models, on the other hand, determine folding of proteins into fibrils and use structural constraints^7–9^. All these methods first require reference data, i.e. a collection of sequences and/or structures of proteins labeled with their ability or inability to form amyloid fibrils. This information is crucial and its imperfection may introduce a bias into prediction methods^10^. However, the process of labeling potential amyloid sequences and confirming the ability to form amyloid fibrils is costly and laborious, usually involving a set of diverse experiments.

Amyloids can be recognized by a characteristic cross-β sheet diffraction pattern observable in X-ray studies. However, to identify the occurrence of an amyloid, less precise methods are usually applied, some of which are direct and others indirect. Direct methods involve microscopy and spectroscopy^11,12^. High resolution microscopic techniques, such as atomic force microscopy (AFM) or transmission electron microscopy (TEM), allow for direct examination of amyloid fibril structures. These methods are focused on their topology and mechanical properties, such as Young modulus^13,14^. Spectroscopic methods involve vibrational spectroscopy^15^, especially IR spectroscopy^16^. In addition to precise information about the kinetics of self-assembly and details about their secondary structures, spectroscopic methods reveal the fraction of amyloid aggregates in the structure.

Indirect techniques rely on the detection (usually through fluorescence) of probes selectively binding to amyloid fibrils. Thioflavin T (ThT) is considered to be the most reliable probe^17^, but Congo Red can also be applied^18^. Although indirect methods are less expensive, there are some concerns regarding their specificity^19^. Therefore, it is helpful if such methods are complemented with direct experimental verification.

As direct and indirect methods focus on different aspects of amyloid fibrils, their results may differ. The problem of experimental validation is further heightened by the elusiveness of amyloid properties^20^. Experimental conditions, such as incubation time, pH and ionic strength, may greatly affect the kinetics of self-assembly, which effectively prevent the development of amyloid fibrils^21^. Therefore, even experimental results bring only partial confidence into the amyloid properties of a peptide or protein.

Such a situation leads to a classical problem of weak labeling (weak supervision)^22^, where some labels (amyloid or non-amyloid) are wrongly assigned to reference instances (proteins or peptides). The weak supervision is common in all applications of machine learning and significantly lowers the performance of a model. Among several approaches proposed to solve this issue, it is suggested to detect mislabeled training data by applying a computational model as a filter, capable of identifying outliers^23^. Here, the outliers are defined as instances predicted computationally with a high probability to have a label opposite to that obtained from a reference dataset. This approach can enhance the classification accuracy achieved by learning algorithms by improving the quality of training data. However, a potential obstacle should be considered, related to overfitting of prediction methods, which may not so easily find mislabeled data in their own training data sets.

To investigate the impact of weak supervision in computational prediction of amyloid proteins, we decided to test AmyloGram, as a filter on training data, which may be mislabeled in databases. The objective was verifying the filtering approach and detecting possible outliers in the learning set. To do this, we selected a subset of peptides for which bioinformatics predictions by AmyloGram were opposite to their labels assigned in experimental AmyLoad and Waltz databases^24,25^. The most extreme outliers, with the highest probability of a predicted label being opposite to that in databases, were then evaluated experimentally. It allowed to verify if the filtering properties of AmyloGram were sufficient to clean the training data from doubtful instances. To strengthen the analysis, we also tested three different bioinformatics predictors of amyloids in this regard. The results revealed how robust are bioinformatics predictors of amyloids to errors in learning datasets.

## Materials and methods

### Data selection

**Figure 1:**
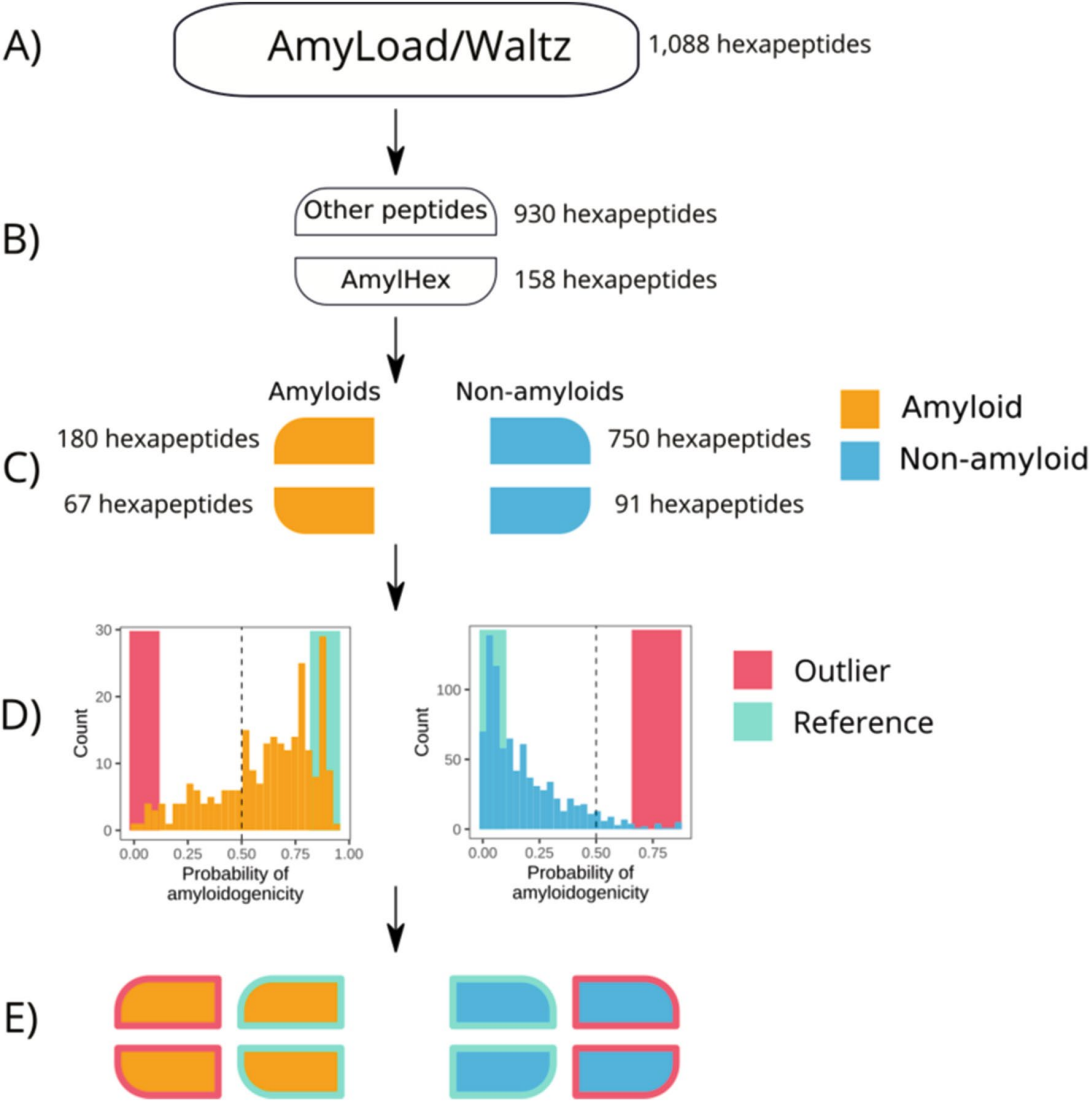
Scheme of peptide selection. (**A**) 1088 hexapeptides in the simplified amino acid alphabet were used to train AmyloGram. (**B**) Two subsets of the sequences were defined. (**C**) Sequences were divided into amyloids and non-amyloids according to their annotations in the database. (**D**) Each peptide was classified with AmyloGram. Peptides with a high probability of classification in agreement with their original annotations were defined as references. Peptides with a high probability of classification opposite to their original annotations were defined as outliers. (**E**) Ten references and 24 outliers were selected for experiments.

Peptides were uploaded from AmyLoad^24^ database. The original dataset used for training AmyloGram included 421 amyloid peptides and 1044 non-amyloid peptides (1465 sequences in total). In terms of their amyloid propensities, all these peptides were also identically annotated in Waltz 2.0 database^25^. The flow chart of the data selection procedure is presented in Fig. 1. First, all sequences with six residues (hexapeptides) and without atypical amino acids were selected. The obtained set included 1088 sequences. It was then divided into two subsets, based on their origin. The first subset contained 158 (67 amyloid and 91 non-amyloid) sequences which were based on the original AmylHex database^26^, and the other set of 930 (180 amyloid and 750 non-amyloid) sequences was based on instances from other sources. AmylHex was the first available data set of amyloid peptides and, although still valuable, it has a strongly biased pattern related to the method by which it was obtained. Therefore, the division in our data processing was introduced to avoid overrepresentation of the AmylHex sequences in the final set and diminish the influence of these biases. Then, all non-redundant amino acid sequences of hexapeptides were converted into the simplified amino acid alphabet obtained in AmyloGram and redundant sequences were removed, leading to 184 encoded amyloid sequences and 683 encoded non-amyloid sequences^4^. Importantly, each of these sequences previously belonged to the reference training dataset and were used to develop AmyloGram.

Since the original experimental annotations do not necessarily have to agree with the classifications obtained with a computational method, the peptides were again classified, now computationally, with AmyloGram (AmyloGram available at: http://www.smorfland.uni.wroc.pl/shiny/AmyloGram/). Peptides that obtained a high probability of classification in agreement with their original database annotations were defined as references. Peptides with a high probability of labels opposite to their original database annotations were defined as outliers. Finally, 10 sequences out of the references were selected and represented with the full amino acid alphabet—we denote this dataset as the *reference dataset*. Similarly, 24 sequences from outliers (represented here with the full amino acid alphabet) were selected and labeled as the *test dataset*. Both sets were used in further experimental validations. The first set served to set up and validate our experimental and chemometric methods, while the other to verify whether the original database annotations of the peptides were correct.

### Materials

All hexapeptide sequences selected for experimental validation were provided by CASLO (CASLO ApS, Denmark). The experiments were carried out on 34 sequences, out of which 10 were *reference* sequences (FNPQGG, FTFIQF, ISFLIF, KPAESD, LVFYQQ, NPQGGY, SFLIFL, TKPAES, YLLYYT, YTVIIE), and 24 were *test* sequences (ALEEYT, ASSSNY, DETVIV, ELNIYQ, FGELFE, FQKQQK, FTPTEK, HGFNQQ, HLFNLT, HSSNNF, MIENIQ, MIHFGN, MMHFGN, NIFNIT, NNSGPN, NTIFVQ, QANKHI, QEMRHF, SHVIIE, STTIIE, STVVIE, SWVIIE, WSFYLL, YYTEFT). The purity of synthesized peptides was in the range between 95% and 99.6%.

### Sample preparation

First, lyophilized hexamers were dissolved and vortexed in 0.1 M NaOH. Next, phosphate-buffered saline (50 mM, pH 7.2) was added to obtain pH = 7. Samples were diluted to the final concentration of 4 mg/ml with Milli-Q water. Then, they were incubated at 37 °C for one month. To assure the reproducibility of new experimental results, reported in this work, the table based on the MIRRAGGE protocol^27^ is available in the Supplement 1, 2, Table 1.

**Table 1:** Reference data set of sequences and their amyloid propensity by different experimental methods ('Yes'—identified as amyloid, 'No'—non-amyloid, 'Yes*'—oligomer, 's'—strong band, 'm'—medium band, 'w'—weak band, 'br'—broad band, 'sh'—shoulder band, band maxima in bold).

No	Sequence	Database	IR microscopy		ATR-FTIR		AFM	Consensus with database annotation
			Amide I [cm^−1^]	Class	Amide I [cm^−1^]	Class	Class	
1	FNPQGG	No	1679(m)/**1641(s)**	No	**1655(s,br)**	No	No	Yes
2	FTFIQF	Yes	1689(m,sh)/**1628(s)**	Yes	1690(w)/**1622(s)**	Yes	Yes*	Yes
3	ISFLIF	Yes	1689(m,sh)/**1631(s)**	Yes	1685(w)/**1631(s)**	Yes	Yes	Yes
4	KPAESD	No	**1665(s,br)**	No	**1678(s,br)/**1640(m,sh)	No	No	Yes
5	LVFYQQ	Yes	**1631(s)**	Yes	1683(w,sh)/**1629(s)**	Yes*	Yes	Yes
6	NPQGGY	No	**1658(s,br)**	No	**1658(s,br)**	No	No	Yes
7	SFLIFL	Yes	1689(m)/**1633(s)**	Yes*	**1632(s)**	Yes	Yes*	Yes
8	TKPAES	No	1652(s,br)	No	**1678(s)/**1640(sh)	No	No	Yes
9	YLLYYT	Yes	1686(m,sh)/**1629(s)**	Yes	1685(m)/**1630(s)**	Yes	Yes*	Yes
10	YTVIIE	Yes	1685(m)/**1627(s)**	Yes	1684(m)/**1626(s)**	Yes	Yes	Yes

The results agree with the original database annotations, which were also in agreement with AmyloGram predictions.

### Experimental evaluation

To keep the experimental validation robust, we employed three direct techniques: two methods of IR spectra measurements and AFM. They complement each other in terms of the presence of aggregates and the exact morphology of fibrils.

#### Atomic force microscopy

AFM images were recorded using Dimension Icon (Bruker) atomic force microscope operating in tapping mode and equipped with a silicon cantilever RTESPA-300 (40 N/m, Bruker), with a typical tip radius of curvature 8 nm. Images (4 × 4, 5 × 5 and 10 × 10 µm^2^) of sample topography were recorded at the resolution of 1024 × 1024 pixels. The scan rate was 0.5–1.0 Hz. In each experiment, 20 µl of peptide solution was deposited on freshly etched mica surface and incubated for 10 min. Subsequently, samples were rinsed with 1 ml of MilliQ water and dried under gentle airflow.

#### Infrared spectroscopy

Two vibrational spectroscopic techniques^28^, commonly used in the field of peptide aggregation, were used in the study: Attenuated Total Reflection—Fourier Transform Infrared (ATR-FTIR)^29^, and Fourier Transform Infrared Microscopy using transmission mode (IR microscopy)^30^. The main drawback of examining proteins in aqueous solutions by means of IR spectroscopy is strong absorbance of water in the region of approximately 1634 cm^-1^^31^. Therefore, in our procedures of spectroscopic measurements we used a dry-film technique^32^.

The ATR-FTIR spectra were collected using a Nicolet 6700 spectrometer (Thermo Scientific, USA) equipped with ATR Accessory with Heated Diamond Top-plate (PIKE Technologies, USA). The spectrometer was continuously purged with dry air. Peptides aliquots of 20 μl volumes were pipetted onto the ATR crystal and allowed to dry out. Spectra were recorded with a resolution of 4 cm^-1^ with 128 co-added scans over the range of 3600–150 cm^-1^, at the constant temperature of 25 °C. The background spectrum was recorded before measurement of the sample spectra using 512 scans under resolution 4 cm^-1^.

The spectra from IR microscopy were recorded using Nicolet iN10 FTIR microscope (Thermo Scientific, USA). Samples were measured with a liquid nitrogen cooled mercury cadmium telluride (MCT-A) detector at the spatial resolution of 10 μm. The microscope was continuously purged with dry air. An area of 450 μm × 450 μm was first selected with the upper aperture (100/5 = 50 μm), then the data were collected. All spectra were recorded in the wave number range from 4000 to 500 cm^-1^; 64 interferograms per sample at the resolution of 4 cm^-1^ were collected. The volume of 10 μl of the solution was applied to barium chloride window cell and allowed to dry out until the coffee-ring was formed^33^. The measurements were carried out at room temperature. For each spectral map the average spectrum was calculated.

Using two IR methods with different acquisition modes allowed us to verify the observations and avoid ambiguity that may arise due to high water absorption^34^. ATR-FTIR spectrophotometer provides one average single spectra obtained from a small area (typically of 3 mm^2^). The FTIR microscopy allows for mapping the probe with a step of 10 μm or less. The liquid nitrogen cooled MCT-A detector is more sensitive and allows to measure smaller aliquots. The built-in camera allows to choose a region of interest, significant for non-homogeneous deposition patterns, created in film techniques. Although IR microscopy is a more precise method and was finally selected as our reference experimental method, we also examined whether ATR-FTIR, which is a cheaper and a more widespread method, would provide different annotations of the peptides.

### Spectroscopic data processing

All spectra were analyzed using the OriginPro 2019 program (OriginLab Corporation, USA). The spectra preprocessing included: baseline correction^35^ and normalization for the Amide I band maximum. The second derivative (DII)^36^ was performed in the range of 1720–1580 cm^-1^ to identify the local maximum of the component bands. The second derivative spectra were smoothed with the Savitzky-Golay filter (parameters: polynomial order 2, window 30)^37^.

### Chemometric analysis

For both types of the IR spectra, Principal Component Analysis (PCA)^38,39^ was performed on DII of the described region, using *PCA* function from scikit-learn Python library^40^ with default parameters.

### Bioinformatics methods

The hexapeptide sequences were classified by bioinformatics methods, such as AmyloGram^4^ (http://www.smorfland.uni.wroc.pl/shiny/AmyloGram/), PATH^41^ (in-house software), FoldAmyloid^6^ (http://bioinfo.protres.ru/fold-amyloid/), and PASTA 2.0^9^ (http://old.protein.bio.unipd.it/pasta2/). AmyloGram is a tool based on machine learning methods, FoldAmyloid and PASTA 2.0 are based on physical models, whereas PATH is our latest method combining physical modeling with machine learning. AmyloGram and PATH were previously trained on the reference peptide sequences, which included all sequences verified here anew (*reference* and *test sets*), using their original annotations in the database. All predictors, excluding PASTA 2.0, were used with their default parameters. In PASTA 2.0, the *peptide* option was chosen to set the thresholds. The presented statistics of classification results included: Accuracy (*Acc*) calculated as the ratio of correctly assigned data labels, Sensitivity (*Sn*) denoting the ratio of correctly identified true positives versus actual positives, and Specificity (*Sp*) meaning the ratio of true negatives versus actual negatives.

## Results

### Experimental verification of the reference dataset of sequences

First, we examined the *reference set*, whose instances had identical annotations in reference databases (AmyLoad and Waltz) and classifications by AmyloGram. The direct microscopy method AFM and two IR methods (ATR-FTIR and IR microscopy) were used to experimentally verify these instances, as well as calibrate our empirical and chemometric methods.

**Figure 2:**
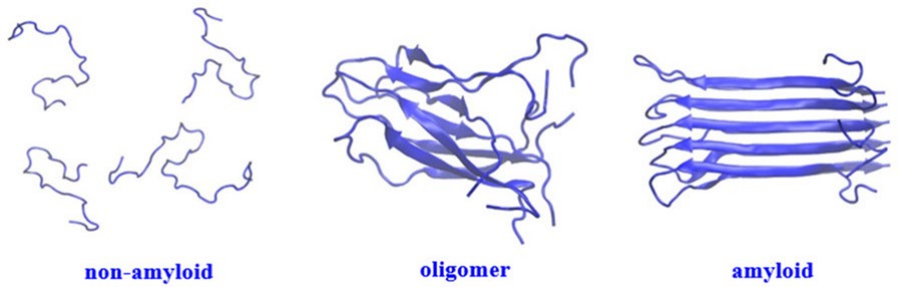
Schemes of peptide classes, representing a general idea.

Based on the AFM micrographs (Supplement 1, 1.1) and spectral characteristics (Supplement 1, 2.1 and 2.2), peptides were annotated into three classes: positive (amyloids), negative (non-amyloids), and oligomers (Fig. 2). The last class is not considered by any bioinformatics method but is evident in experimental analyses and may pose a problem for computational tools in its correct classification.

**Figure 3:**
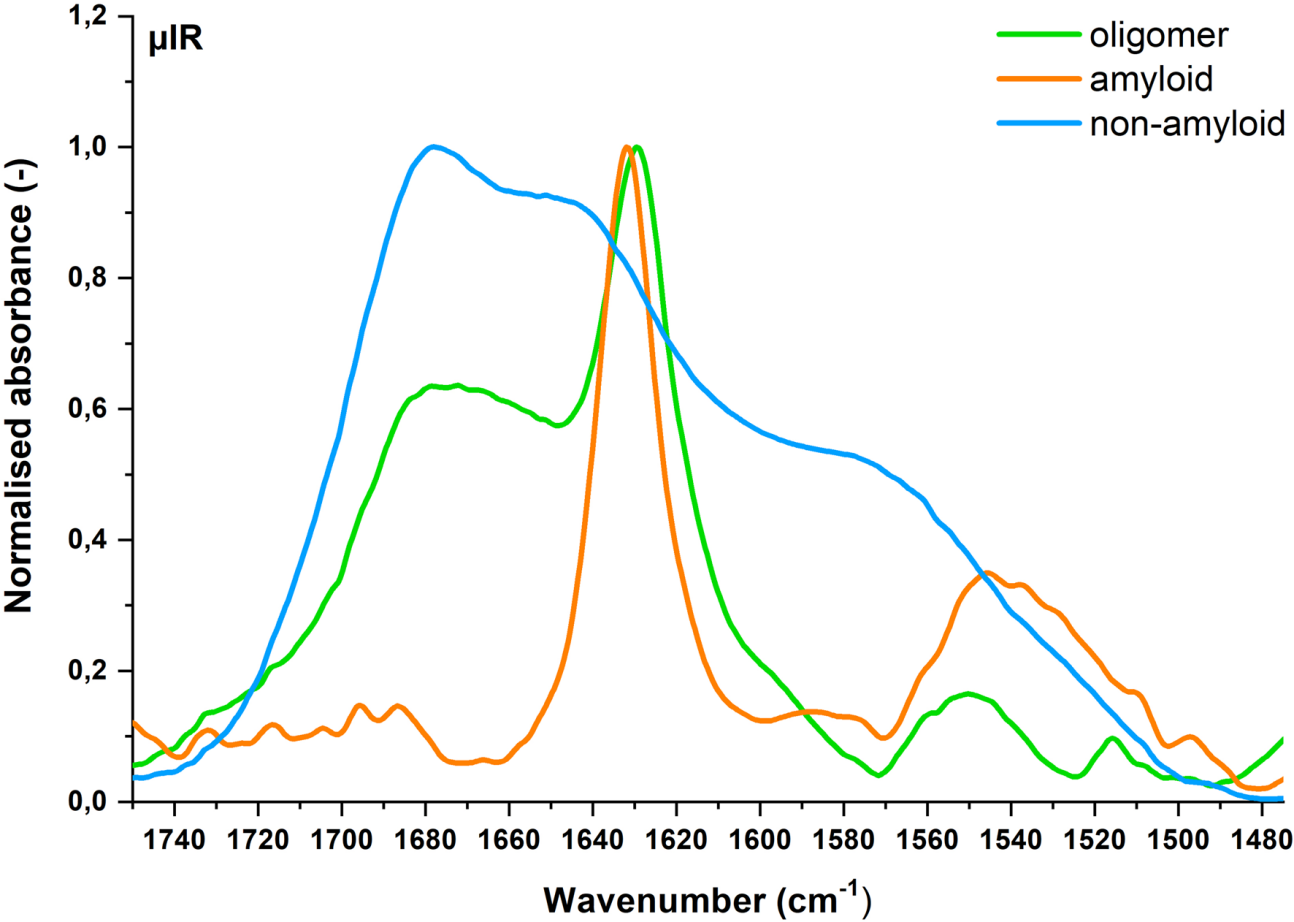
Representative IR microscopy spectra: amyloid (LVFYQQ) in red, oligomer (SFLIFL) in green, non-amyloid (KPAESD) in blue.

The IR spectra can be fairly easily analyzed in terms of potential amyloidogenicity of the peptides, showing different characteristics for non-amyloids, small assemblies of amyloid aggregates known as oligomers, and mature fibrils. Exemplary spectra of our *reference set*, representing each of these classes, are presented in Fig. 3.

Amide bands characteristic of peptide bonds dominate in the protein infrared spectra. The most intensive, Amide I, occurs in the range of 1700–1600 cm^-1^, which corresponds to C = O stretching vibrations^34^. Amyloid fibrils show absorbance between 1611 and 1630 cm^-1^, usually close to 1630 cm^-1^, while for native β-sheet proteins it extends from 1630 to 1643 cm^-1^. This method also enables recognition of typical amyloid oligomers, indicated by the presence of two local maxima in Amide I region. The major one is located at 1630 cm^-1^, and the minor peak, resulting from a strong dipolar coupling, ranges between 1695 and 1685 cm^-1^. The latter peak is often approximately five-fold weaker than the absorption at 1630 cm^-1^ (Fig. 3)^29,35,36^.

Both IR methods, used in our studies, provided compatible results. As expected, they were in general agreement with their original annotations in the databases (Table 1). However, there were differences, which may have resulted from the experimental specifics (see Materials and Methods), or the oligomer class. The sequence SFLIFL provided slightly different spectra in both IR methods: transmission (microscopy) and attenuated reflection (ATR-FTIR) (Table 1 and Supplement 1, 2.4, Table 7), indicating formation of oligomers which did not transform into fibrils.

The differences may be caused by the artifacts incited by the thickness of the sample—thicker samples can raise the spectrum in the transmission mode in IR microscopy. On the other hand, the signal registered with ATR-FTIR could be influenced by water molecules in contact with the crystal^42^. The contact of peptide molecules with the diamond surface in ATR-FTIR can accelerate the aggregation process. Therefore, IR microscopy could be regarded as a more accurate experimental method. The study confirmed that infrared spectroscopy could be used as a time-efficient tool to investigate the formation of different types of aggregates.

**Figure 4:**
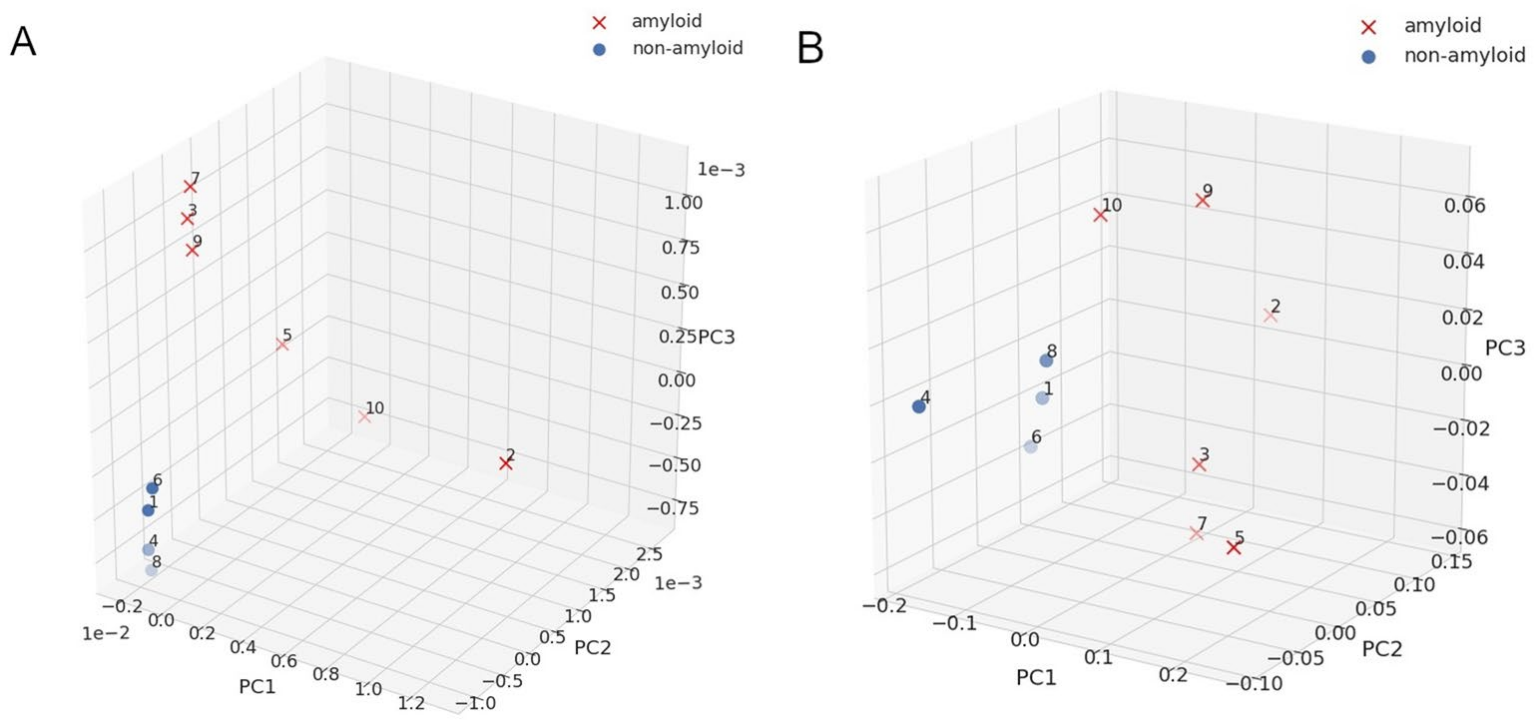
PCA plots for IR spectra of the *reference set*: (**A**) ATR-FTIR. (**B**) IR microscopy. Crosses denote amyloids and dots represent non-amyloids, as identified on the spectra by a human expert.

Furthermore, for fast and more robust identification of amyloids and non-amyloids, we applied principal component analysis (PCA) on the IR spectra^38,39^. PCA separated out 4 sequences in the ATR-FTIR spectra of the *reference set*: NPQGGY, FNPQGG, KPAESD, TKPAES. All these sequences were identified as non-amyloids by a human expert based on different experimental methods. Each of the remaining sequences, more dispersed in the plot, was previously identified either as an amyloid or oligomer—based on the same experimental methods. Similarly, PCA for IR microscopy spectra also distinguished the group of non-amyloid peptides (Figs. 4A,B).

**Figure 5:**
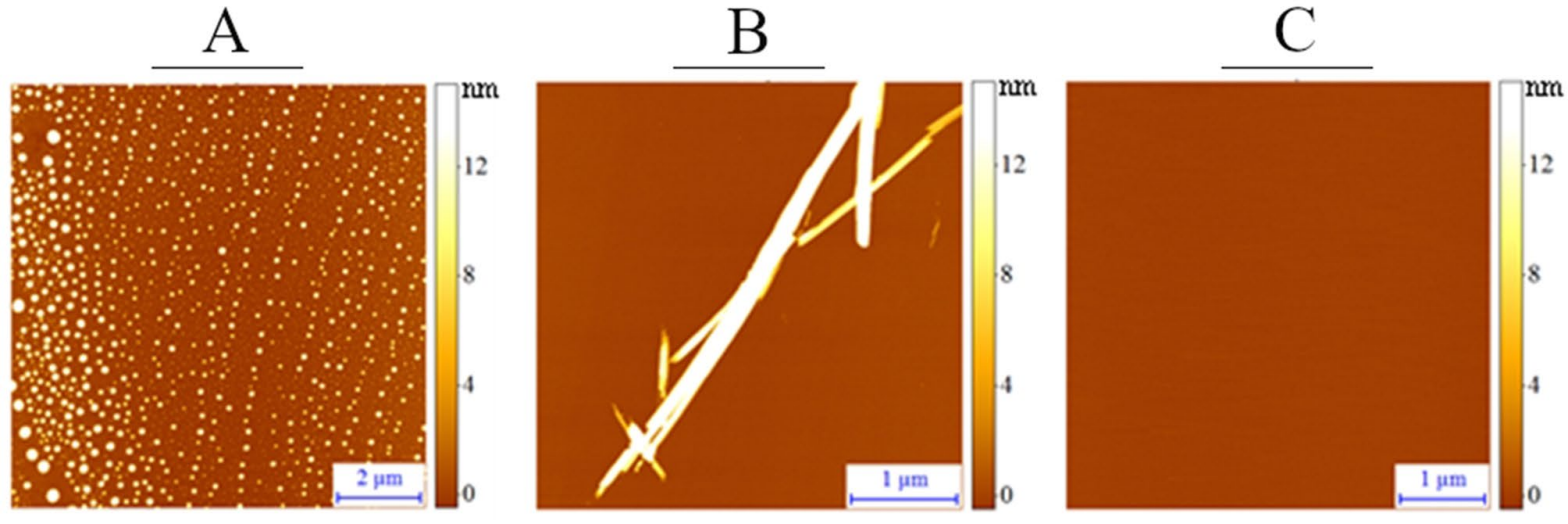
Representative AFM micrographs: (**A**) oligomer (FTFIQF), (**B**) amyloid (LVFYQQ), C. non-amyloid (NPQGGY).

The results obtained by means of IR spectroscopy were verified with high resolution microscopy using AFM (Fig. 5, Supplement 1, 2.1, Table 2). In these studies, the process of hexapeptide self-assemblance was observed a few minutes after preparation of the peptide solution.

**Table 2:** Reference sequences and their amyloid propensity obtained by different bioinformatic methods, compared to IR microscopy ('Yes'—amyloid, 'No'—non-amyloid, 'Yes*'—oligomer).

No	Sequence	IR microscopy	AmyloGram	FoldAmyloid	PASTA 2.0	PATH (LR)	PATH (RF)	Consensus with IR (%)
1	FNPQGG	No	No	No	No	No	No	100
2	FTFIQF	Yes	Yes	Yes	No	Yes	Yes	80
3	ISFLIF	Yes	Yes	Yes	Yes	Yes	Yes	100
4	KPAESD	No	No	No	No	No	No	100
5	LVFYQQ	Yes	Yes	Yes	No	Yes	Yes	80
6	NPQGGY	No	No	No	No	No	No	100
7	SFLIFL	Yes*	Yes	Yes	Yes	Yes	Yes	100
8	TKPAES	No	No	No	No	No	No	100
9	YLLYYT	Yes	Yes	Yes	No	Yes	Yes	80
10	YTVIIE	Yes	Yes	Yes	Yes	No	Yes	80

### Bioinformatics analysis of the reference dataset

The annotations based on IR microscopy results were compared with all bioinformatics methods, including not only AmyloGram, but also FoldAmyloid, PASTA 2.0 and PATH (Table 2). Generally, all methods recognized the sequences correctly and in agreement with IR spectroscopy. Concurrence of the IR microscopy and computational results was at a high level, reaching 75 or 100%. We want to emphasize that due to the very small size of the set and the method of its selection (based on the strong prediction probabilities by AmyloGram), the prediction results from different bioinformatics methods by no means should be treated as benchmarks of their individual general performances.

### Annotations of sequences in the test dataset

The experiments on the *reference dataset* showed that IR spectroscopy is in good agreement with much more laborious and expensive AFM method. Therefore, IR spectroscopy was selected for experimental validation of the annotations in the *test set*, which was the main objective of our studies. The results obtained for 24 sequences that constituted this set are presented in Table 3. These data did not take into account the component bands from aromatic amino acids, such as: phenylalanine (1600), tyrosine (1616) and tryptophan (1620)^43^.

**Table 3:** Test sequences and their amyloid propensities ('Yes'—identified as amyloid, 'No'—non-amyloid, 'Yes*'—oligomer, 's'—strong band, 'm'—medium band, 'w'—weak band, 'br'—broad band, 'sh'—shoulder band, band maxima in bold), compared with the original database annotation (all in disagreement with AmyloGram predictions).

No	Sequence	Database	IR microscopy		ATR-FTIR		Consensus with database annotation
			Amide I [cm^−1^]	Class	Amide I [cm^−1^]	Class	
1	ALEEYT	Yes	**1655(s,br)**	No	**1654(s)**	No	No
2	ASSSNY	Yes	**1649(m,sh)**	No	**1655(m,br)**	No	No
3	DETVIV	No	1685(w)/**1635(s)**	Yes*	1685(m)/**1633(s)**	Yes*	No
4	ELNIYQ	No	1661(w,sh)/**1635(s**)	No	1681(m,br)/1668(m,br)/**1635(s)**	No	Yes
5	FGELFE	No	**1660(s)/**1650(w)	No	**1659(s)**	No	Yes
6	FQKQQK	No	**1660(s,br)**	No	**1682(s,br)**	No	Yes
7	FTPTEK	No	**1660(s,br)**	No	**1680(s,br)**	No	Yes
8	HGFNQQ	Yes	**1662(s,br)**	No	**1682(s,br)**	No	No
9	HLFNLT	Yes	**1674(s,br)**	No	**1680(s,br)/**1633(m,br)	No	No
10	HSSNNF	Yes	**1649(m,br)**	No	**1680(s)/**1646(m,sh)	No	No
11	MIENIQ	Yes	**1656(s,br)**	No	**1655(s,br)**	No	No
12	MIHFGN	Yes	**1677(s,br)**	No	**1680(s,br)**/1646(m,br)	NO	NO
13	MMHFGN	Yes	**1675(s)**	No	**1676(s,br)**	No	No
14	NIFNIT	Yes	**1657(s)**	No	**1663(s,br)**	No	No
15	NNSGPN	Yes	1676(sh)/**1648(s,br)**	No	**1676(s,br)/**1654(m,br)	No	No
16	NTIFVQ	No	**1629(s)**	Yes	1682(w)/**1631(s)**	Yes*	No
17	QANKHI	Yes	**1680(s,br)**	No	**1681(s)/**1653(sh)	No	No
18	QEMRHF	Yes	**1679(s,br)**	No	**1676(s,br)/**1655(sh)	No	No
19	SHVIIE	No	1688(m)/**1630(s)**	Yes	1684(m)/**1633(s)**	Yes	No
20	STTIIE	No	**1657(s,br)**	No	1681(m)/**1630(s)**	Yes*	Yes ambiguous
21	STVVIE	No	1685(w,br)/**1633(s)**	Yes	1682(w,br)/**1630(s)**	Yes*	NO
22	SWVIIE	No	1682(w,sh)/**1631(s)**	Yes	1684(w)/**1631(s)**	Yes	No
23	WSFYLL	No	**1658(s,br)**	No	1675(w,sh)/**1637(s)**	No	Yes
24	YYTEFT	No	**1665(s,br)**	No	**1659(s,br)**	No	Yes

Out of 24 hexapeptides, only one peptide, STTIIE, gave an ambiguous result in terms of IR spectroscopic methods (Table 3 and Supplement 1, 3.2, Table 12). For STTIIE, we observed in IR microscopy two local maxima, 1657 cm^-1^ corresponding to the strong band from α- helix and 1607 cm^-1^ assigned to tyrosine vibrations. Therefore, this peptide was labeled as non-amyloid. Although Amide I band is very broad, there are many component bands, which are confirmed by the second derivative (Supplement 1, 3.1.2.2., Table 11). This fact cannot exclude that the oligomerization process could have occurred. However, based on the ATR-FTIR, this structure can be identified as oligomer, therefore in terms of classification by bioinformatics tools—positively. Two local maxima characteristic of oligomers can be observed in the spectrum. The first maximum at 1684 cm^-1^ and the second, more intense, at 1633 cm^-1^ (Supplement 1, 3.2). The spectral features can be assigned to anti-parallel oligomeric β-sheets. For the remaining 23 sequences both IR techniques provided consistent results.

Based on the results presented in Table 4, we observed that in the *test set*, for which AmyloGram’s classification disagreed with the original database annotations, 17 (71%) peptides were indeed misannotated, 12 (70%) of them were false positives and 5 (30%) were false negatives. In the set of misannotated sequences, five were actually amyloids and all of them (100%) were misannotated, while 19 were non-amyloids and 12 (63%) of them were misannotated. A variety of reasons could have contributed to it, which is shown in Supplement 2, Table 1.

**Table 4:** Test sequences and their amyloid propensities predicted by different bioinformatics methods and compared with IR microscopy ('Yes'—amyloid, 'No'—non-amyloid, 'Yes*'—oligomer).

No	Sequence	Database	IR microscopy	AmyloGram	PATH (LR)	PATH (RF)	FoldAmyloid	PASTA 2.0	Bioinformatics consensus with IR [%]
1	ALEEYT	Yes	No	No	No	No	No	No	100
2	ASSSNY	Yes	No	No	No	No	No	No	100
3	DETVIV	No	Yes*	Yes	No	Yes	No	Yes	60
4	ELNIYQ	No	No	Yes	No	No	Yes	No	60
5	FGELFE	No	No	Yes	No	No	No	No	80
6	FQKQQK	No	No	Yes	No	No	No	No	80
7	FTPTEK	No	No	Yes	No	No	No	No	80
8	HGFNQQ	Yes	No	No	No	No	No	No	100
9	HLFNLT	Yes	No	No	No	Yes	Yes	No	60
10	HSSNNF	Yes	No	No	No	No	No	No	100
11	MIENIQ	Yes	No	No	No	No	No	No	100
12	MIHFGN	Yes	No	No	No	No	No	No	100
13	MMHFGN	Yes	No	No	No	No	No	No	100
14	NIFNIT	Yes	No	No	No	Yes	Yes	No	60
15	NNSGPN	Yes	No	No	No	No	No	No	100
16	NTIFVQ	No	Yes	YES	Yes	Yes	Yes	No	80
17	QANKHI	Yes	No	No	No	No	No	No	100
18	QEMRHF	Yes	No	No	No	No	No	No	100
19	SHVIIE	No	Yes	Yes	No	No	Yes	Yes	60
20	STTIIE	No	No	Yes	No	No	No	No	80
21	STVVIE	No	Yes	Yes	No	Yes	Yes	Yes	80
22	SWVIIE	No	Yes	Yes	No	Yes	Yes	Yes	80
23	WSFYLL	No	No	Yes	Yes	Yes	Yes	No	80
24	YYTEFT	No	no	Yes	No	No	No	No	80

For comparison, the '*Database*' column presents original annotations from the databases.

Importantly, all these sequences were previously used for training of AmyloGram, using the misannotated labels. However, AmyloGram was capable of recognizing misannotated instances in its training dataset, which showed its robustness with regard to incorrect labeling. Only 7 sequences out of this set were correctly annotated in the database and misclassified by AmyloGram. The majority of them were sequences rich in aromatic and charged amino acids.

**Figure 6:**
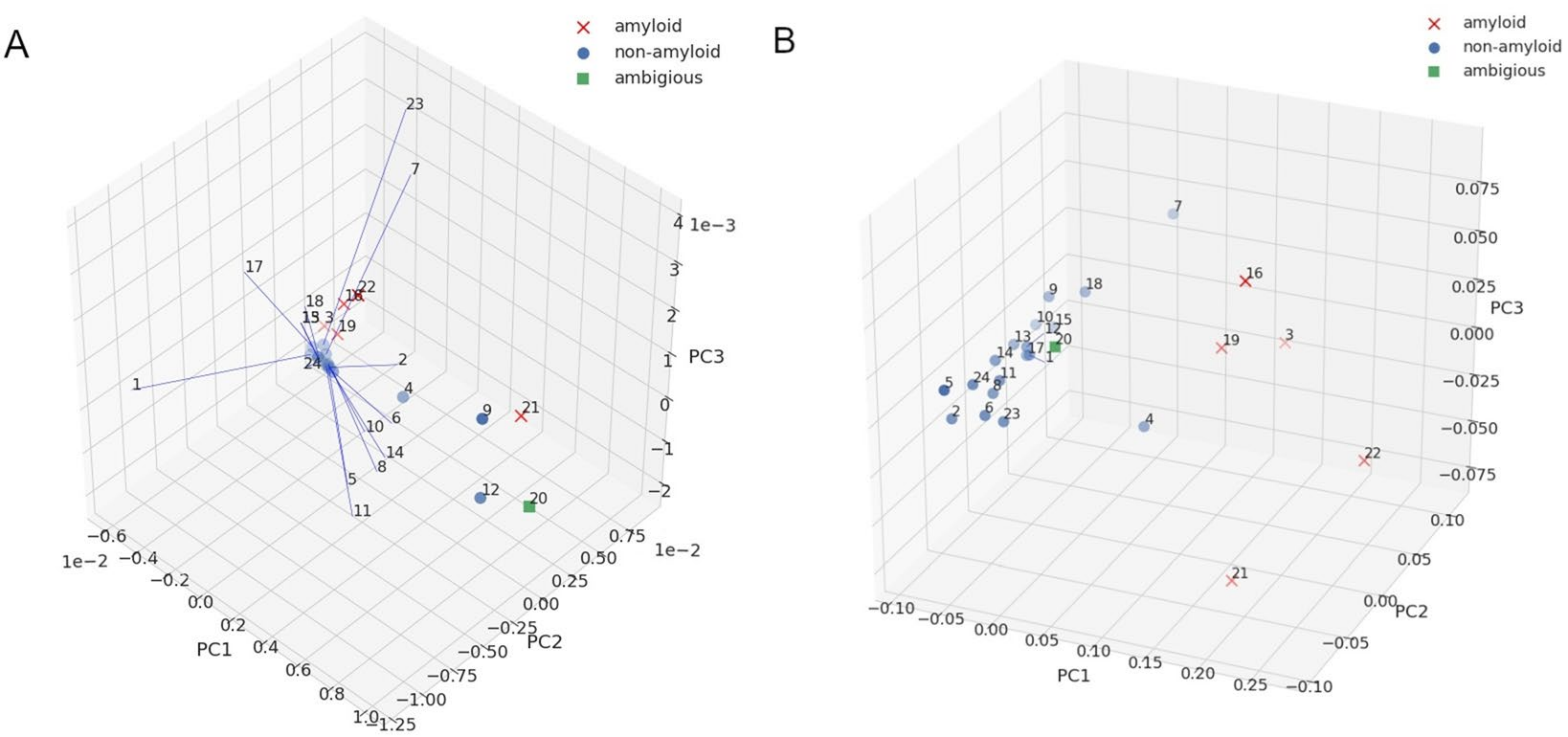
PCA plots for IR spectra of the *test set*: (**A**) ATR-FTIR. (**B**) IR microscopy. Crosses denote amyloids and dots represent non-amyloids, as identified on the spectra by a human expert.

IR spectra of the *test set* were analyzed with PCA. Similar to the *reference set*, a good separation between amyloids and non-amyloids (as previously identified by the human expert) was obtained for majority of the sequences (Fig. 6), especially good agreement was obtained for the data from IR microscopy (Fig. 6B). The automated PCA analysis on the spectra from ATR-FTIR located the sequence no 20 (STTIIE), which was ambiguous with regard to IR experiments, outside the amyloid and non-amyloid clusters. As expected, PCA based on the spectra from the IR microscopy assigned it to the cluster of non-amyloids. A few other sequences were also located outside the aggregated clusters, either in the PCA analysis on ATR-FTIR or IR microscopy, but there was no overlap between them, except the sequence no 4 (ELNIYQ). Interestingly, although this sequence was experimentally verified as non-amyloid, it was predicted by AmyloGram and FoldAmyloid as a potential amyloid.

The annotations from IR microscopy for the *test set* were compared with results from other bioinformatics predictors, out of which PATH is another method also trained on the set including the misannotated sequences, which can use either logistic regression (LR) or random forest (RF) classification methods. Except for AmyloGram and PATH, other bioinformatics methods might have not been trained on the misannotated data (methods not developed in our group). The majority of methods agreed with our IR results (Table 4, detailed scores in Supplement 2: Table 2 and Table 3), including the cases in which the original annotation in the database was contradicted by the experiments presented in Table 3. There were a few less obvious instances. For example, the consensus between bioinformatics methods dropped for two sequences: DETVIV and ELNIYQ. In case of DETVIV, the IR microscopy result was also ambiguous—it showed oligomeric rather than fibril aggregates. In case of ELNIYQ, PCA-based classification of the spectra did not locate it in the cluster of non-amyloids. The bioinformatics analysis identified the sequence no 20 (STTIIE), which was ambiguous regarding IR experiments, as non-amyloid (3 out of 4 methods), which agrees with IR microscopy and associated PCA analysis. AmyloGram was the only method which misclassified it as amyloid. Table 5 presents aggregated results of the bioinformatics analysis.

**Table 5:** Consensus between annotations obtained from bioinformatics methods and IR microscopy (Accuracy *Acc*, Sensitivity *Sn*, Specificity *Sp*). Presented results are for: (A) all 24 sequences from the *test set*, (B) only 17 sequences from the *test set*, which turned out misannotated in databases.

	AmyloGram			PATH (LR)			PATH (RF)			FoldAmyloid			PASTA 2.0		
	Acc	Sn	Sp	Acc	Sn	Sp	Acc	Sn	Sp	Acc	Sn	Sp	Acc	Sn	Sp
A	0.71	1	0.63	0.79	0.2	0.95	0.83	0.8	0.84	0.79	0.8	0.79	0.92	0.8	1
B	1	1	1	0.76	0.2	1	0.82	0.8	0.83	0.82	0.8	0.83	0.94	0.8	1

All computational methods correctly identified the majority of misannotated sequences. Again, we want to emphasize that due to the size of the set and the method of its selection (based on the strong adverse predictions by AmyloGram), the prediction results from different bioinformatics methods should not be treated as benchmarks of their general performances.

## Discussion

Amyloid aggregates may lead to serious health problems, when peptides enter the amyloid pathway, therefore it is crucial to recognize them correctly and identify specific sequence features, which can be associated with amyloidogenicity. Although several direct and indirect experimental methods are available to determine the amyloid propensity of a sequence, all of them are laborious and expensive. What is even more important, the results of the experiments are not always conclusive and identical, if obtained with different experimental methods. This may lead to misannotation of the sequences regarding their amyloidogenicity. Moreover, errors occurring in databases, related to data retrieval or curation, may additionally contribute to mislabeling of the data.

Many bioinformatics methods have been developed to classify amyloidogenicity of amino acid sequences. These methods readily and efficiently support experiments, saving time and money. However, all computational methods, like modeling in general, heavily depend on the data used in the model construction. Data including misannotated instances may lead to an incorrect model, not even revealed by standard evaluation methods, which would also rely on the mislabeled reference data.

Therefore, we posed a question: How robust could be bioinformatics methods to the problem of certain misannotations in the reference data? The problem occurred when we observed that some of the computational classifications did not always agree with labeling of the reference training data. To address the question, we selected a set of sequences and tested their amyloidogenicity by experimental and computational methods. The first part of the set, when classified by our predictor AmyloGram, strongly agreed with the initial labeling in the database, as it was expected. We used it to set up our experimental and chemometric methods, including two IR spectroscopy methods, ATR-FTIR and IR microscopy, and AFM microscopy. The second part of the set included sequences whose classification by AmyloGram strongly disagreed with the initial labeling in the reference databases. Besides amyloids and non-amyloids, we also noted that a third class of structures, i.e. oligomers, should be included in the analyses.

As a result, we observed that 17 out of 24 non-compatible sequences were actually misannotated in the original databases. Therefore, the bioinformatics predictor proved resistant to overfitting, and able to find errors in its own training data. Tests on other bioinformatics predictors showed that all of them were able to classify the misannotated data correctly, with accuracies reaching at least 80% or more—also for methods which were trained on all these mislabeled data. This proves that bioinformatics methods can be successfully applied to evaluate quality of experimental data and used for their filtering. However, we underline that the fraction of mislabeled instances cannot be excessively high in the training set.

## Supplementary Information

Supplementary Information 1.

Supplementary Information 2.
