# Supplementary material for: Bioinformatics methods for identification of amyloidogenic peptides show robustness to misannotated training data
Source: Sci Rep. 2021 Apr 26;11:8934. doi: 10.1038/s41598-021-86530-6 (PMC8076271; doi:10.1038/s41598-021-86530-6)
Supplement: Supplementary file 1 — Supplementary Information 1. [file 41598_2021_86530_MOESM1_ESM.pdf]

Supplement 1

Bioinformatic methods for identification of amyloidogenic peptides shows robustness to misannotated training data

Natalia Szulc[1,2], Michal Burdukiewicz[3,4 ξ], Marlena Gąsior-Głogowska[1], Jakub W. Wojciechowski[1], Jaroslaw Chilimoniuk[5], Paweł Mackiewicz[5], Tomas Šneideris[6], Vytautas Smirnovas[6], Malgorzata Kotulska[1* ξ]

[1]Wroclaw University of Science and Technology, Department of Biomedical Engineering, Wybrzeże Stanisława Wyspiańskiego 27, 50-370 Wroclaw, Poland

[2]University of Lorraine, CNRS, LPCT, F-54000 Nancy, France

[3]Medical University of Bialystok, Jana Kilinskiego 1, 15-089 Białystok, Poland

[4]Institute of Biochemistry and Biophysics, Polish Academy Sciences, Adolfa Pawińskiego 5A, 02-106 Warsaw, Poland

[5]University of Wroclaw, Faculty of Biotechnology, 51-148 Wroclaw, Poland

[6]Vilnius University, Life Sciences Center, Institute of Biotechnology, Vilnius, LT-10257, Lithuania

Table of Contents

1. The MIRRAGGE table 1
2. Reference dataset 2
2.1. Atomic force microscopy (AFM) 2
2.2. Vibrational spectroscopy 4
2.2.1. Attenuated Total Reflection–Fourier Transform Infra-Red (ATR-FTIR) 4
2.2.2. Scheme of the experiment 4
2.2.3. Normalized spectra in the range of 3600-1000 cm^{-1} 4
2.2.4. Amide spectra with second derivative 7
2.3. Infrared Microscopy using transmission mode (IR microscopy) 11
2.3.1. Scheme of the experiment 11
2.3.2. Normalized spectra in the range of 3600-1000 cm^{-1} 12
2.3.3. Amide spectra with second derivative 14
2.4. IR microscopy vs ATR-FTIR 20
2.5. PCA analysis 22
3. Test dataset 24
3.1. Vibrational spectroscopy 24
3.1.1. Attenuated Total Reflection–Fourier Transform Infra-Red (ATR-FTIR) 24
3.1.1.1. Normalized spectra in the range of 3600-1000 cm-1 24
3.1.1.2. Amide spectra with second derivative 30
3.1.2. Infrared Microscopy using transmission mode (IR microscopy) 42
3.1.2.1. Normalized spectra in the range of 3600-1000 cm^{-1} 42
3.1.2.2. Amide spectra with second derivative 47
3.2. IR microscopy vs ATR-FTIR 59
3.3. PCA analysis 64

1. The MIRRAGGE table

Table 1 MIRRAGGE – Minimum Information Required for Reproducible AGGregation Experiments

**Sample details**					
Organism/ Peptide Sequence	UniProt code (residues)	pI	GRAVY	Extinction coefficient [A280, 0.1%(w/v)]*	MW from chemical composition (Da)
**FNPQGG**	ERF3_YEAST, (92-97)	14	-1.1	23300	617.65
**FTFIQF**	RO60_HUMAN, (126-131)	14	1.45	40100	800.94
**ISFLIF**	PRIO_HUMAN, (244-249)	14	2.93	31100	737.93
**KPAESD**	ERF3_YEAST, (164-169)	6.7	-1.92	13900	644.67
**LVFYQQ**	ALMS1_HUMAN, (817-822)	9.8	0.42	29380	795.92
**NPQGGY**	ERF3_YEAST,(74-79)	9.4	-1.78	20780	633.65
**SFLIFL**	PRIO_HUMAN, (245-250)	14	2.82	31100	737.93
**TKPAES**	ERF3_YEAST, (163-168)	9.8	-1.45	13900	630.69
**YLLYYT**	B2MG_HUMAN, (83-88)	9.3	0.5	32140	833.97
**YTVIIE**	N.A.	6.6	1.28	19980	735.87
**ALEEYT**	APOA1_HUMAN, (256-261)	4.2	-0.56	19980	723.77
**ASSSNY**	RNAS1_BOVIN, (46-51)	9.9	-0.9	20380	626.62
**DETVIV**	FLO1_YEAST, (306-311)	3.9	0.86	13900	673.76
**ELNIYQ**	CSGA_ECOLI, (44-49)	6.8	-0.58	20780	777.86
**FGELFE**	N.A.	4.2	0.33	31100	739.81
**FQKQQK**	ERF3_YEAST, (129-134)	14	-2.58	23700	804.94
**FTPTEK**	B2MG_HUMAN, (90-95)	9.9	-1.27	22500	720.81
**HGFNQQ**	FACE1_HUMAN, (153-158)	14	-1.88	28900	728.76
**HLFNLT**	FUT9_HUMAN, (150-155)	14	0.5	28100	742.87
**HSSNNF**	N.A.	14	-1.5	28500	703.7
**MIENIQ**	N.A.	6.6	0.06	16530	745.89
**MIHFGN**	PRIO_MOUSE, (137-142)	14	0.35	29930	716.85
**MMHFGN**	PRIO_MESAU, (138-143)	14	-0.08	31760	734.89
**NIFNIT**	N.A.	14	0.68	23300	719.83
**NNSGPN**	CSGA_ECOLI, (37-42)	14	-2.22	15100	600.58
**NTIFVQ**	FUS_HUMAN, (285-290)	14	0.63	23300	719.83
**QANKHI**	N.A.	14	-1.3	19900	708.81
**QEMRHF**	N.A.	11	-1.67	31280	845.97
**SHVIIE**	N.A.	7.6	0.95	19100	695.81
**STTIIE**	N.A.	6.6	0.55	13900	661.75
**STVVIE**	N.A.	6.6	1.32	13900	645.75
Source (supplier, catalogue No. or reference)				CASLO ApS (Scion Denmark Technical University)	
N-terminal modification				----	
C-terminal modification				----	
Internal modifications				----	
Other modifcations				----	
Purity (%)				≥95%	
**Purification** (If applicable)	Chromatography techniques			----	
	Concentration of stock solution (M, mg/mL)			----	
	Storage/Reconstitution buffer			----	
	Method of protein quantification			----	
	Storage conditions			Lyophilized	
	Additional key information			----	
**Sample quality control**					
**Polishing step**	Immediately before the aggregation assay			----	
	Concentration (M, mg/mL)			----	
	Method quantification			----	
**Aggregation assay**	Method of detection			ATR-FTIR; µIR	
	Equipment details			Nicolet 6700 spectrometer (Thermo Scientific, USA) equipped with ATR Accessory with Heated Diamond Top-plate	

			(PIKE Technologies, USA); FTIR Nicolet iN10 (Thermo Scientific, USA)
	Measurement parameters		128 scans, 4 cm^{-1}, T= 25 ºC;
	Plate/cuvette reference		-----
	Assay volume		10 µL
	Evaporation control method		----
	Seeding details (if applicable)		----
	Shaking	Intensity	----
		Shaking mode	----
		Frequency	----
	Beads	Reference	----
		Number/assay	----
	Temperature (ºC)		37
	Concentration (M, mg/mL)		4 mg/mL
	Aggregation buffer and aditives		0.1 M NaOH, 50 mM PBS, pH 7.4
	Measurement frequency		----
	Assay duration		2h
	Plate/cuvette setup		----
	Additional key steps		----
* Calculated based on: http://bestsel.elte.hu/extcoeff.php [Extinction coefficient at 205 nm, concentration units: $M^{-1}cm^{-1}$			

2. Reference dataset

2.1. Atomic force microscopy (AFM)

Table 2 AFM images

No.	Sequence	AFM
1	FNPQGG	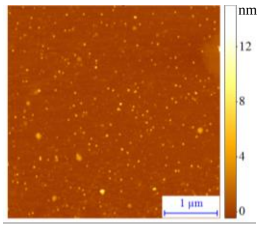
2	FTFIQF	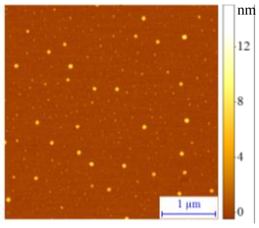
3	ISFLIF	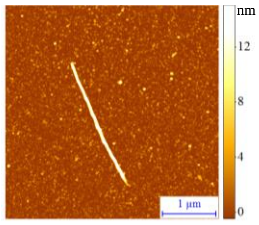

4	KPAESD	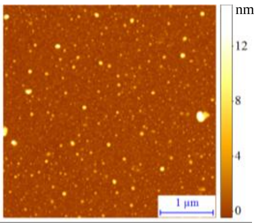
5	LVFYQQ	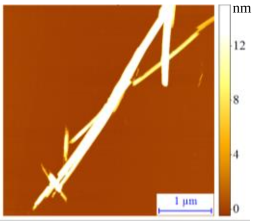
6	NPQGGY	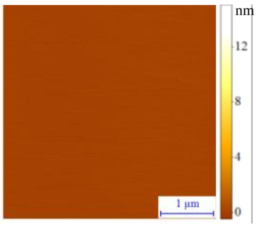
7	SFLIFL	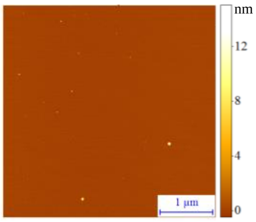
8	TKPAES	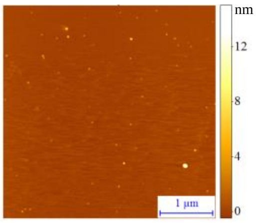
9	YLLYYT	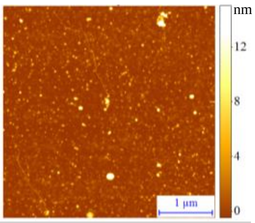
10	YTVIIE	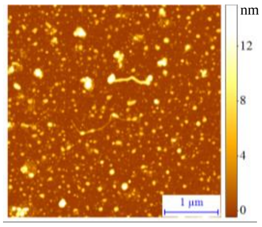

2.2. Vibrational spectroscopy

2.2.1. Attenuated Total Reflection–Fourier Transform Infra-Red (ATR-FTIR)

2.2.2. Scheme of the experiment

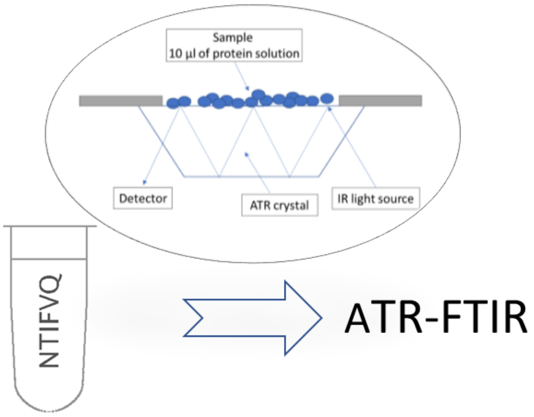

2.2.3. Normalized spectra in the range of 3600-1000 cm^{-1}

Table 3 All spectra of examined hexapeptides

No.	Sequence	ATR-FTIR
1	FNPQGG	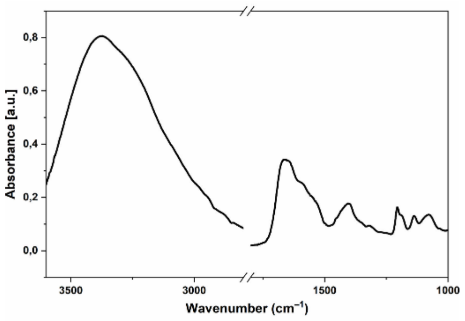
2	FTFIQF	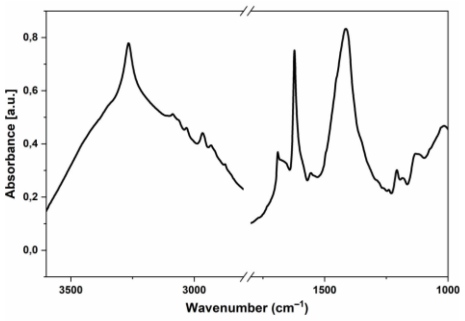

3	ISFLIF	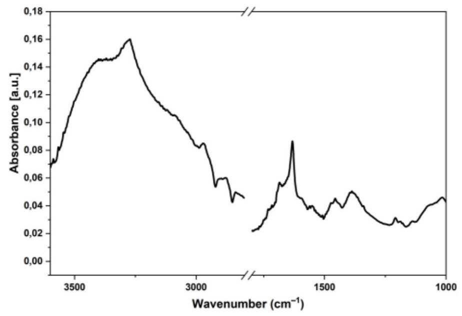
4	KPAESD	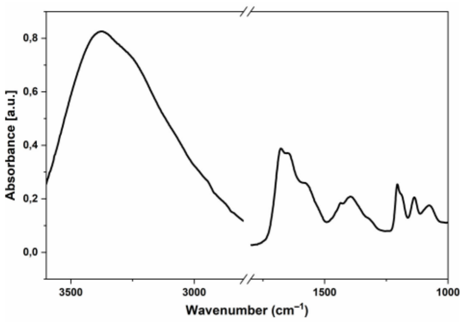
5	LVFYQQ	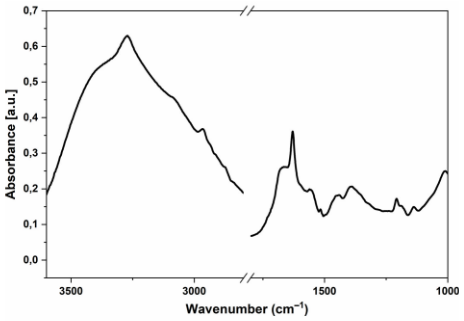
6	NPQGGY	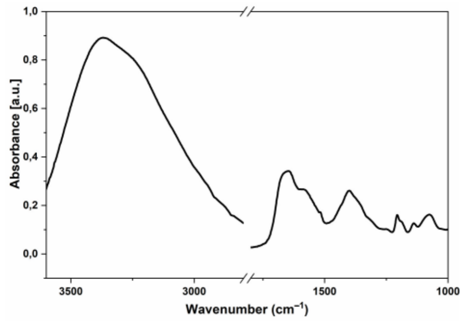
7	SFLIFL	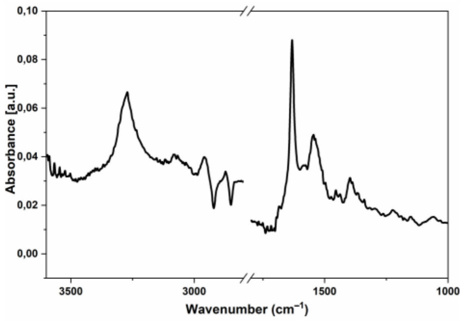

8	TKPAES	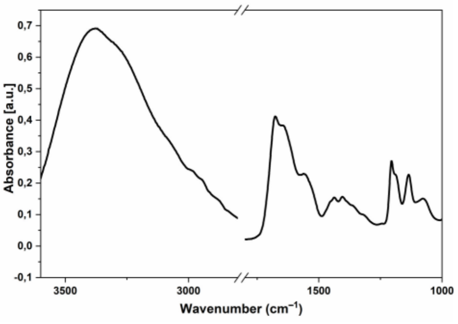
9	YLLYYT	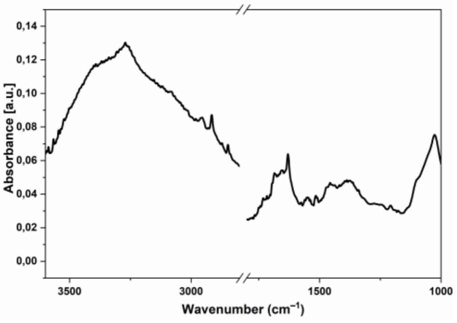
10	YTVIIE	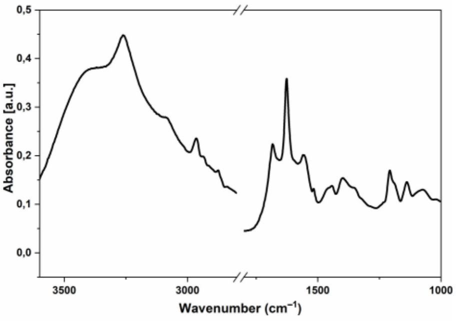

2.2.4. Amide spectra with second derivative

Table 4 Amide I spectrum with second derivative

<table>
<tr><th>No.</th><th>Sequence</th><th>ATR-FTIR</th></tr>
<tr><td>1</td><td>FNPQGG</td><td>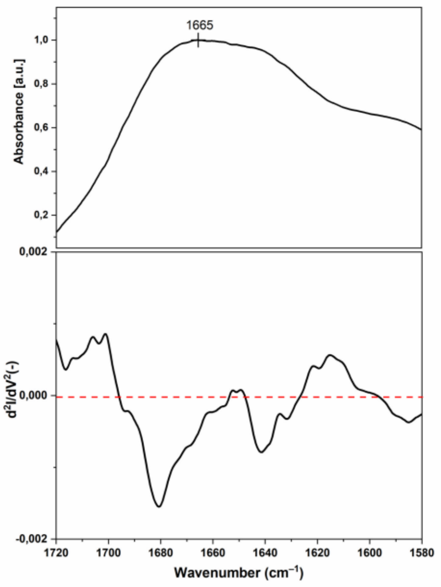
</td></tr>
<tr><td>2</td><td>FTFIQF</td><td>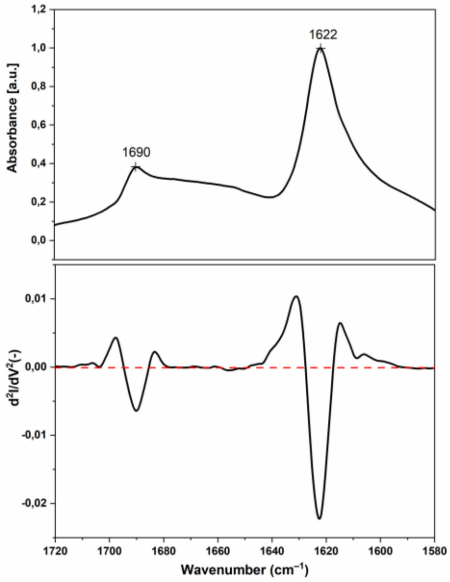
</td></tr>
</table>

<table>
<tr><td>3</td><td>ISFLIF</td><td>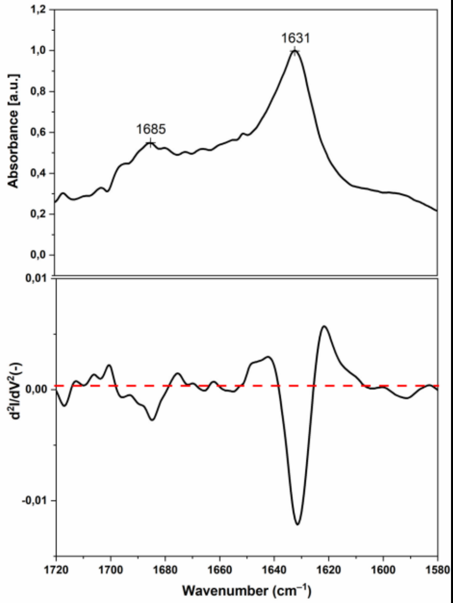</td></tr>
<tr><td>4</td><td>KPAESD</td><td>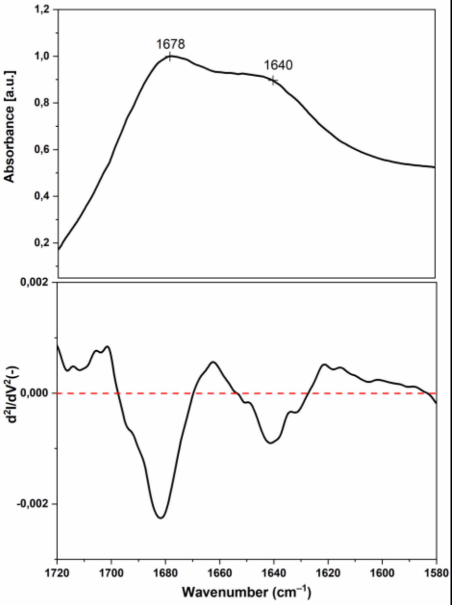</td></tr>
</table>

5	LVFYQQ	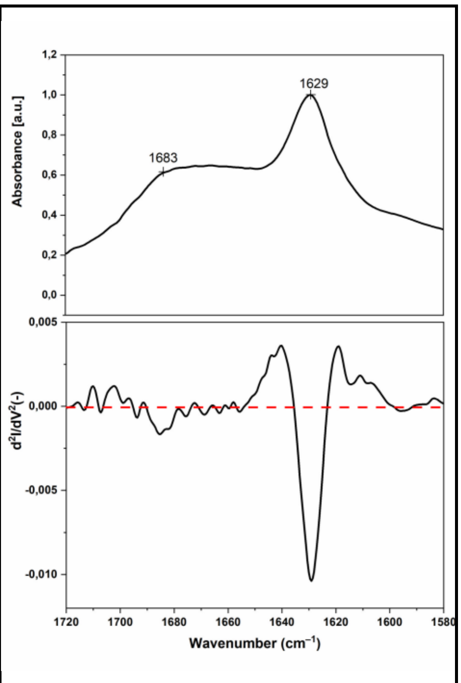
6	NPQGGY	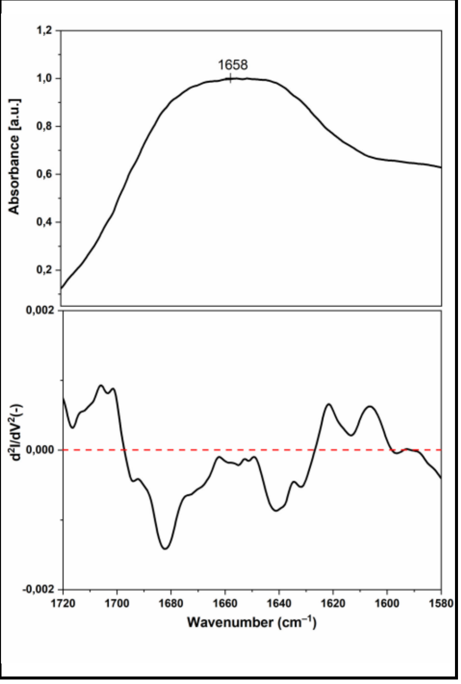

7	SFLIFL	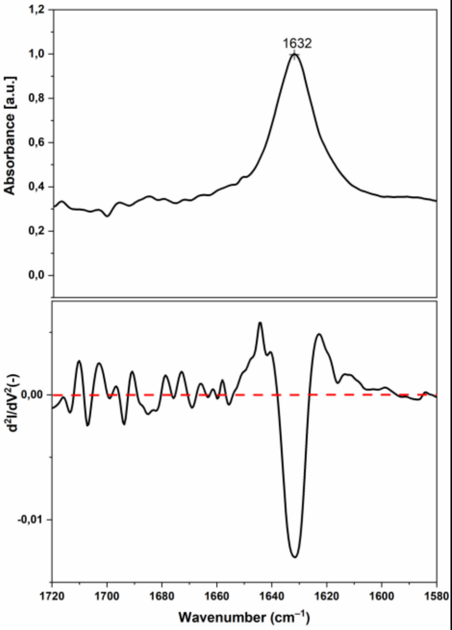
8	TKPAES	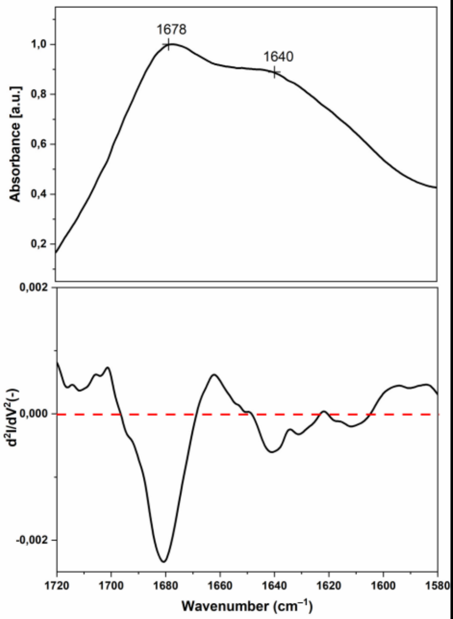

<table>
<tr><td>9</td><td>YLLYYT</td><td>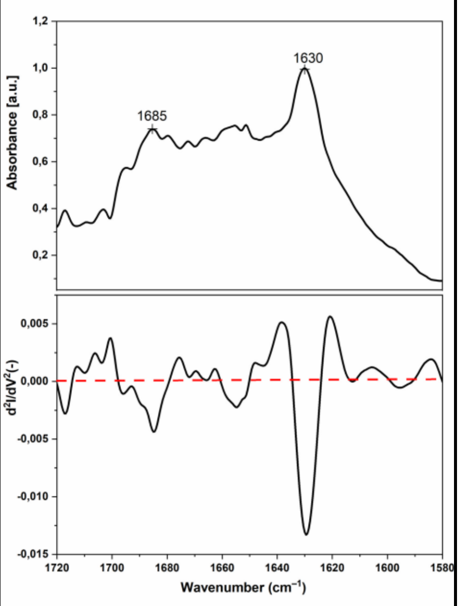
</td></tr>
<tr><td>10</td><td>YTVIIE</td><td>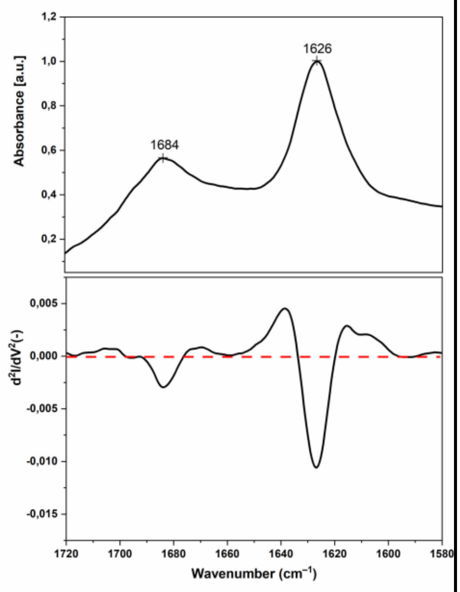
</td></tr>
</table>

2.3. Infrared Microscopy using transmission mode (IR microscopy)

2.3.1. Scheme of the experiment

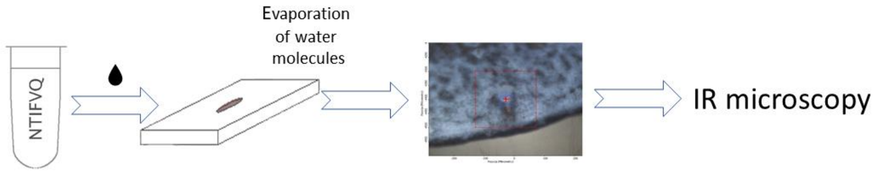

2.3.2. Normalized spectra in the range of 3600-1000 cm^{-1}

Table 5 All spectra of examined hexapeptides

No.	Sequence	FTIR microscopy
1	FNPQGG	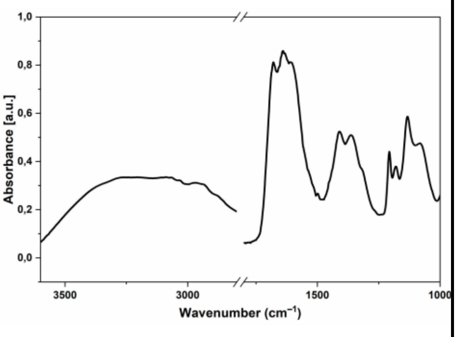
2	FTFIQF	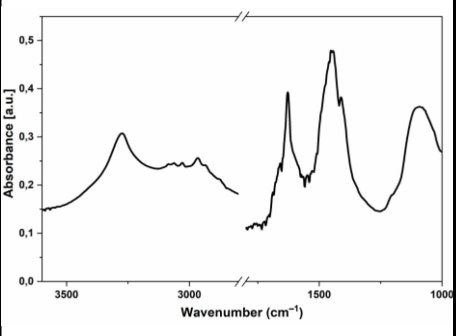
3	ISFLIF	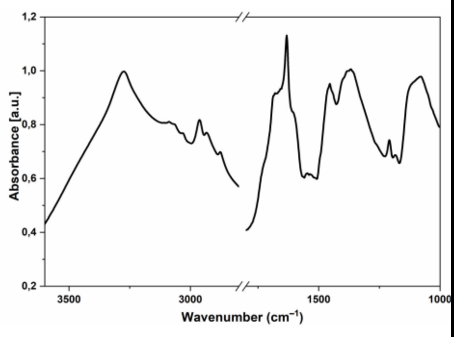
4	KPAESD	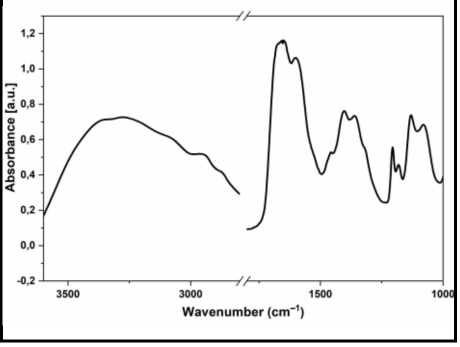

5	LVFYQQ	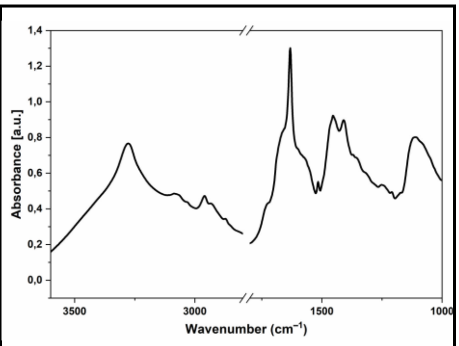
6	NPQGGY	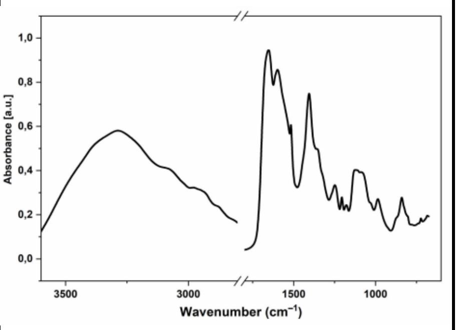
7	SFLIFL	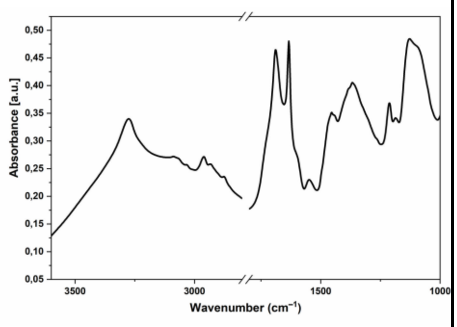
8	TKPAES	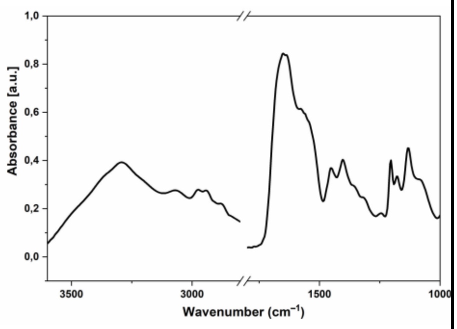
9	YLLYYT	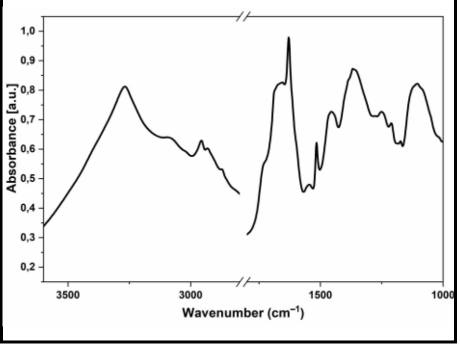

10	YTVIIE	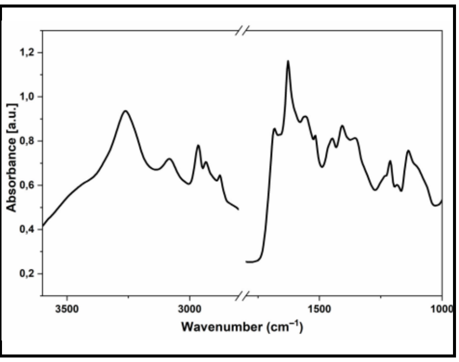

2.3.3. Amide spectra with second derivative

Table 6 Amide I spectrum with second derivative

No.	Sequence	FTIR microscopy
1	FNPQGG	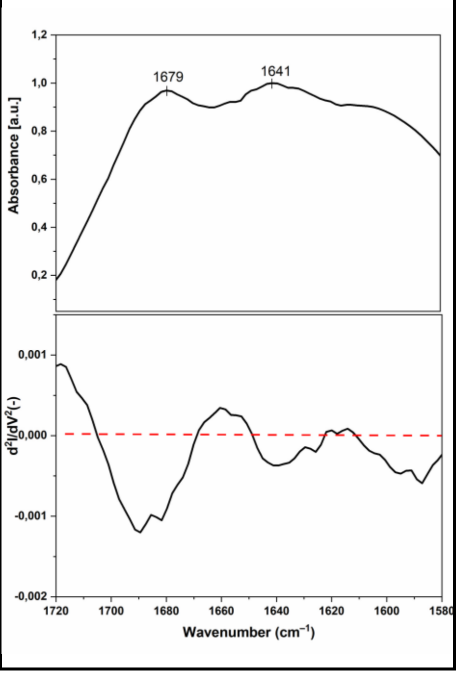

2	FTFIQF	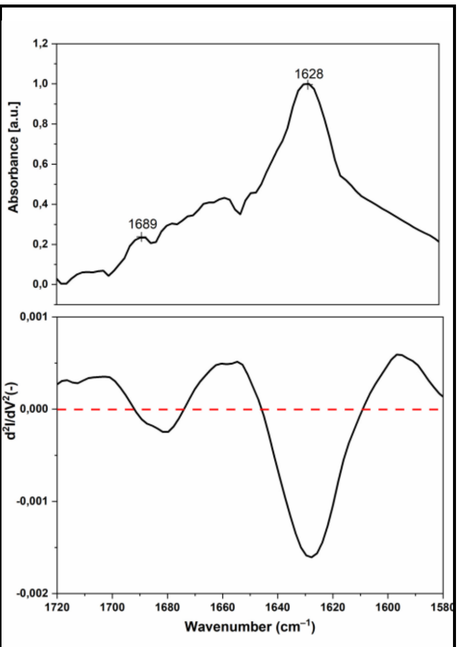
3	ISFLIF	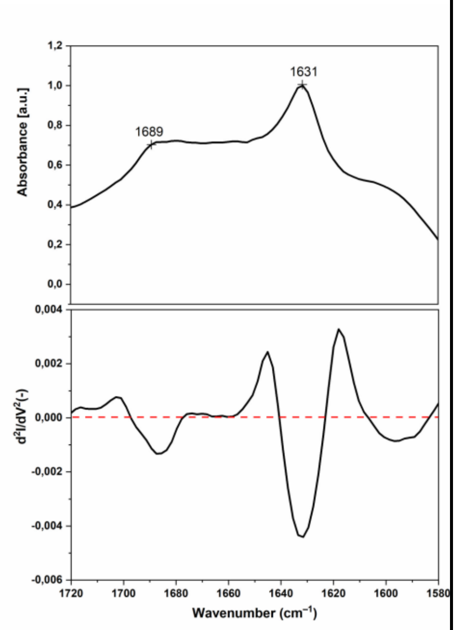

4	KPAESD	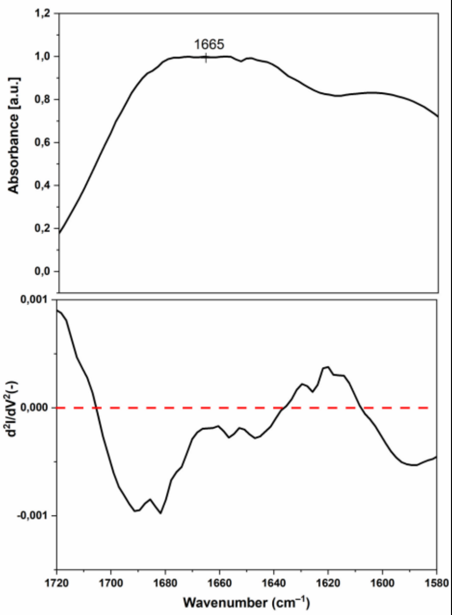
5	LVFYQQ	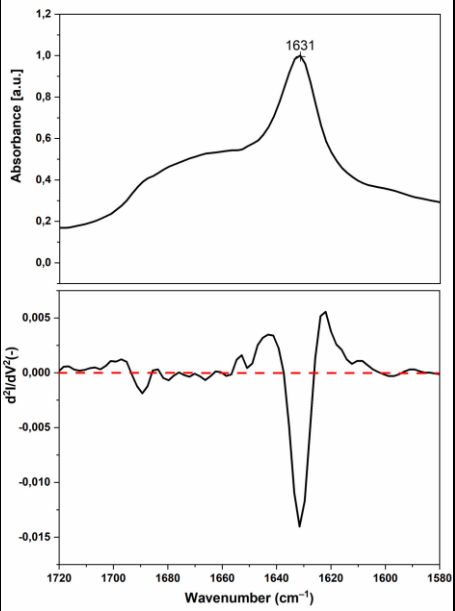

6	NPQGGY	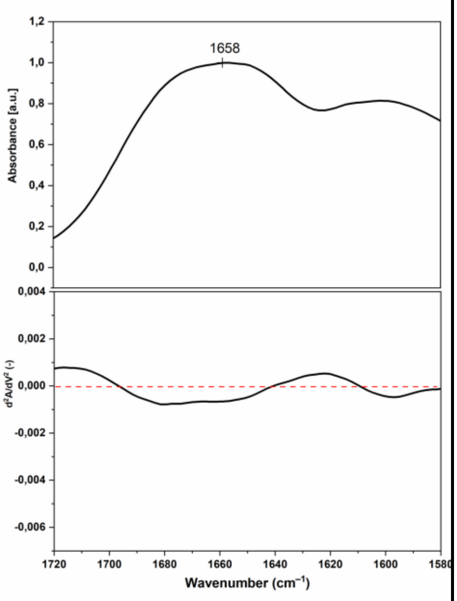
7	SFLIFL	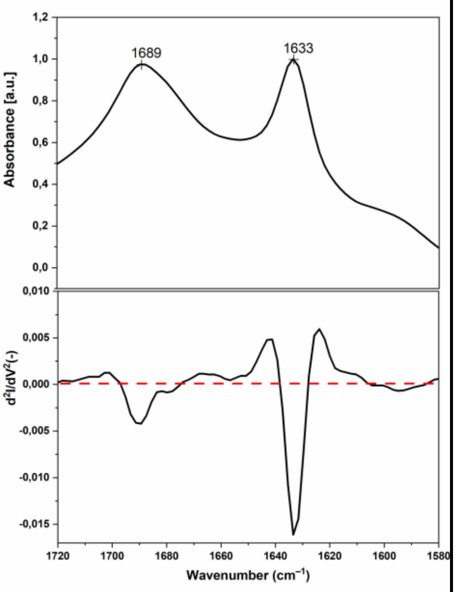

8	TKPAES	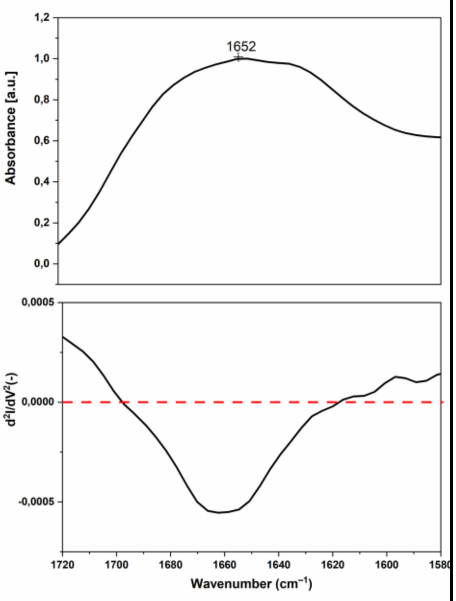
9	YLLYYT	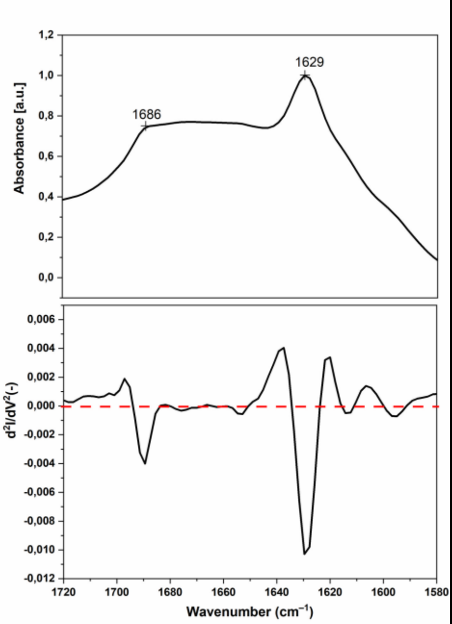

10	YTVIIE	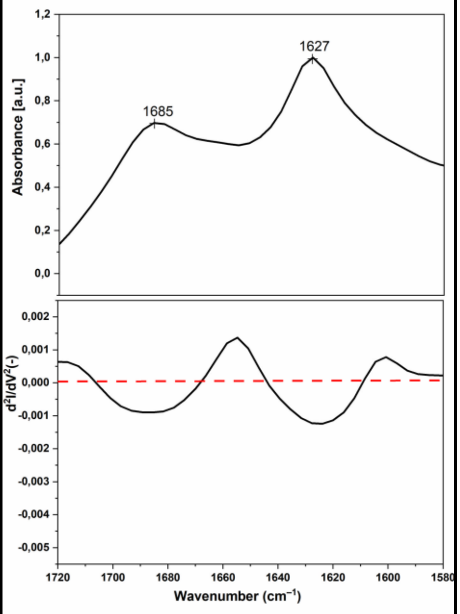

2.4. IR microscopy vs ATR-FTIR

Table 7 Differences between FTIR (transmission mode) and ATR-FTIR spectra of examined hexapeptides

No.	Sequence	IR microscopy vs ATR-FTIR
1	FNPQGG	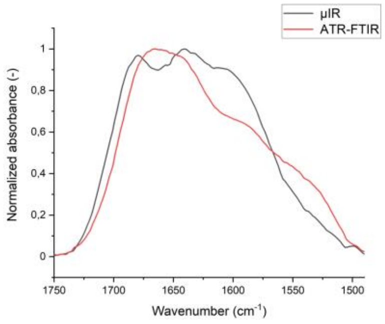
2	FTFIQF	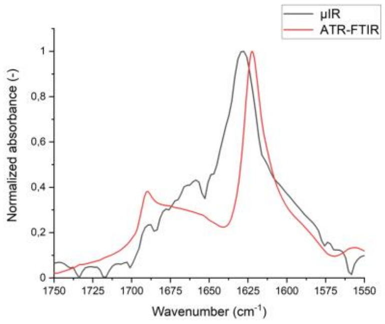
3	ISFLIF	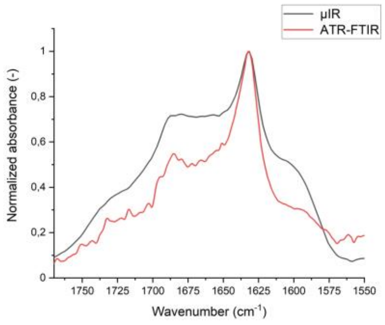
4	KPAESD	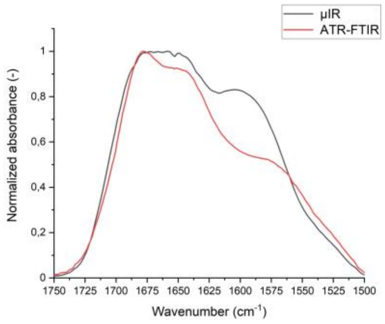

5	LVFYQQ	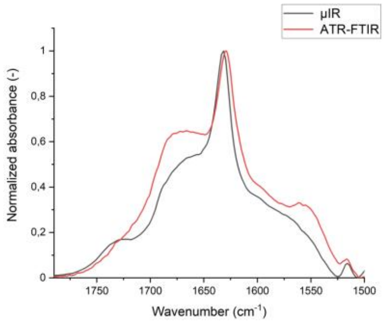
6	NPQGGY	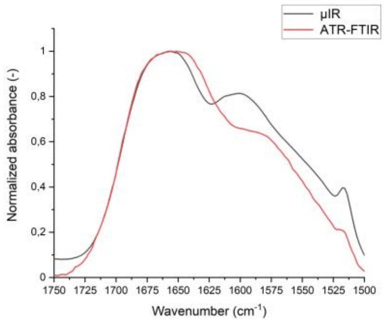
7	SFLIFL	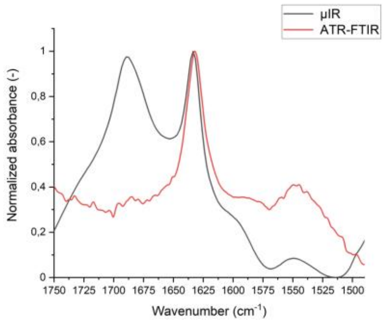
8	TKPAES	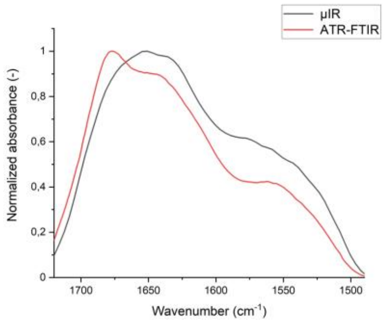

9	YLLYYT	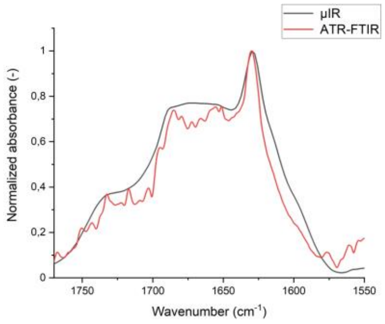
10	YTVIIE	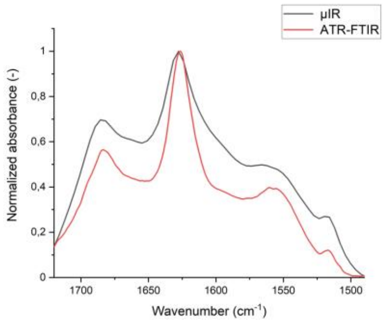

2.5. PCA analysis

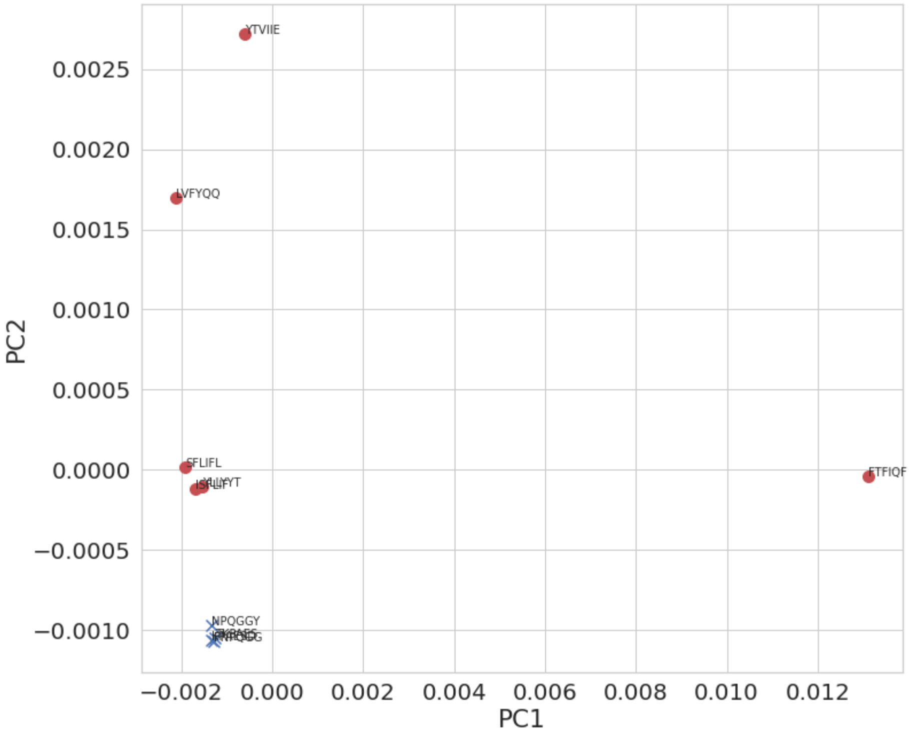

Figure 1 PCA plot for ATR-FTIR spectra of the reference set. Red dot assigned to amyloid and blue cross to non-amyloid.

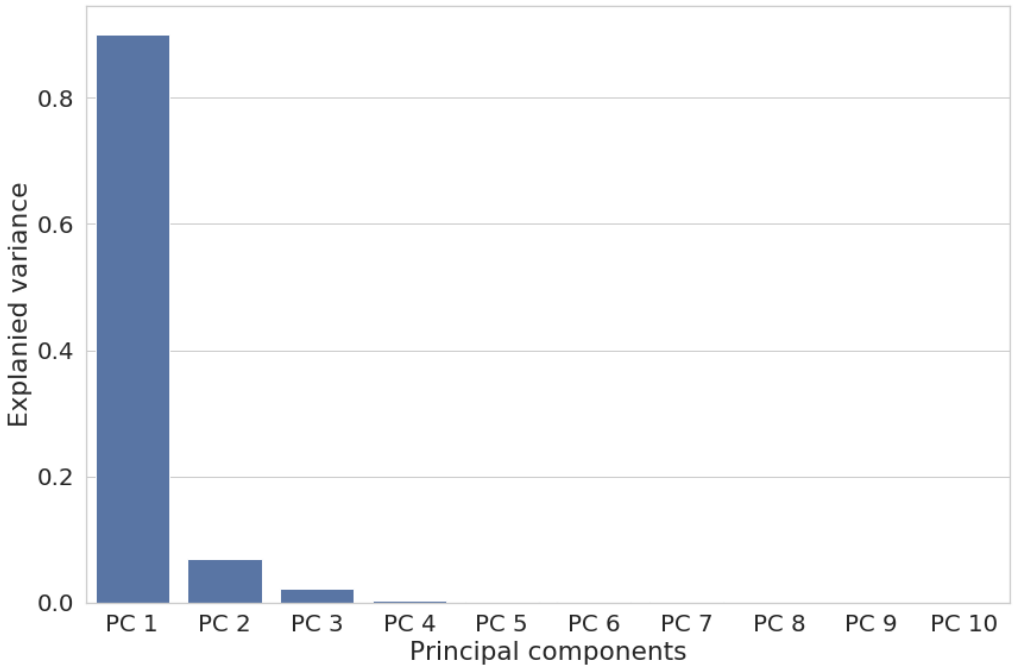

Figure 2 The distribution of principal components for ATR-FTIR spectra in the reference set.

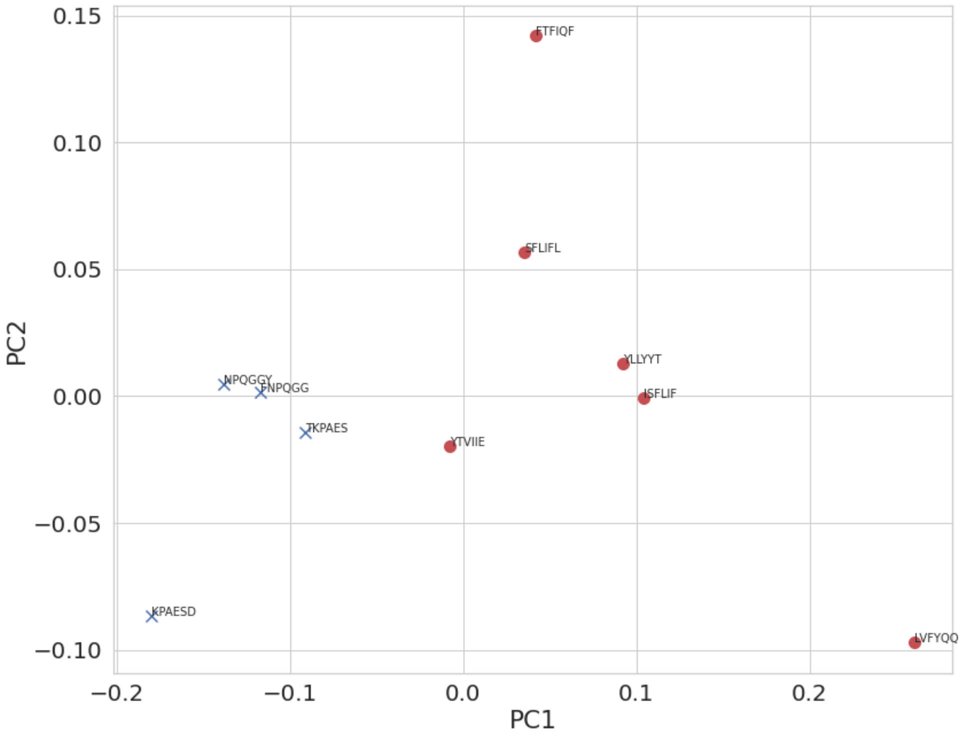

Figure 3 PCA plot for IR microscopy spectra of the reference set. Red dot assigned to amyloid and blue cross to non-amyloid.

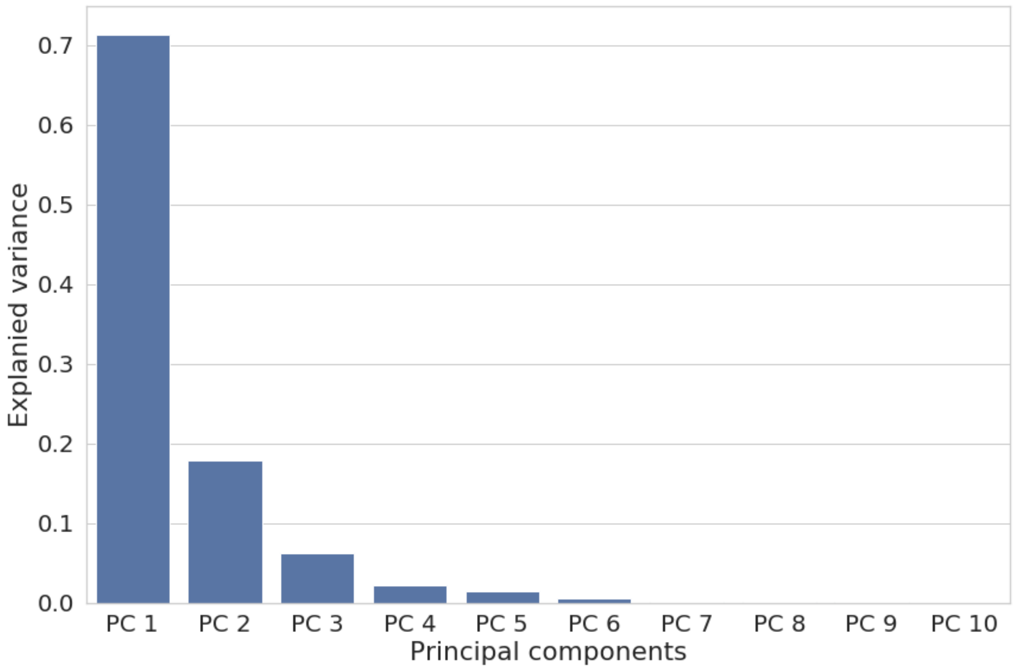

Figure 4 The distribution of principal components for IR microscopy spectra in reference set.

3. Test dataset

   3.1. Vibrational spectroscopy

      3.1.1. Attenuated Total Reflection–Fourier Transform Infra-Red (ATR-FTIR)

         3.1.1.1. Normalized spectra in the range of 3600-1000 cm-1

Table 8 All spectra of examined hexapeptides

No.	Sequence	ATR-FTIR
1	ALEEYT	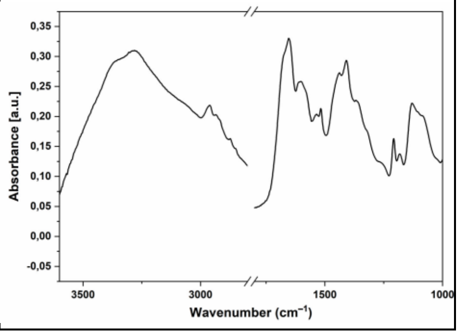

2	ASSSNY	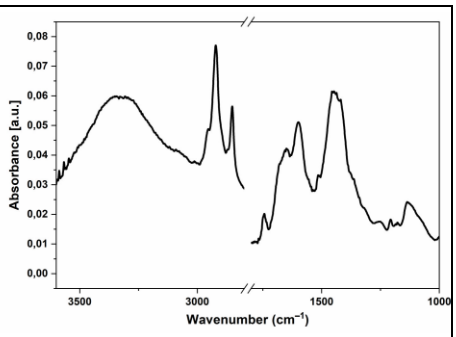
3	DETVIV	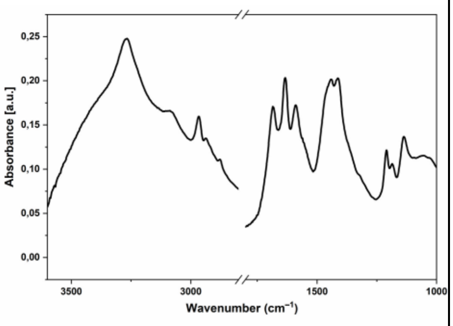
4	ELNIYQ	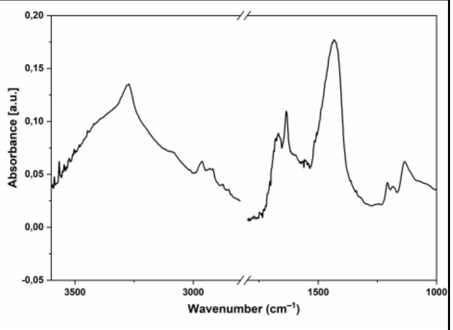
5	FGELFE	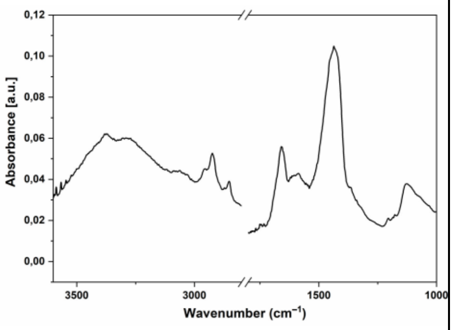
6	FQKQQK	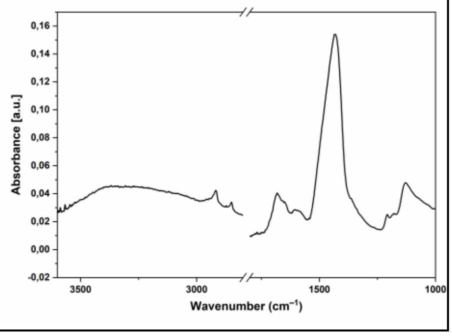

7	FTPTEK	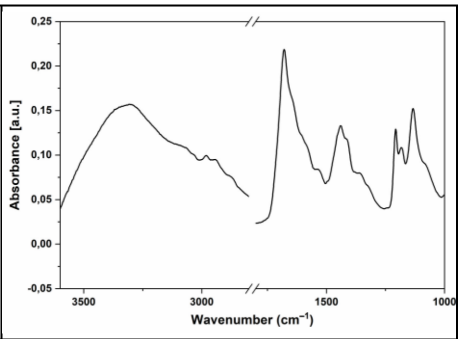
8	HGFNQQ	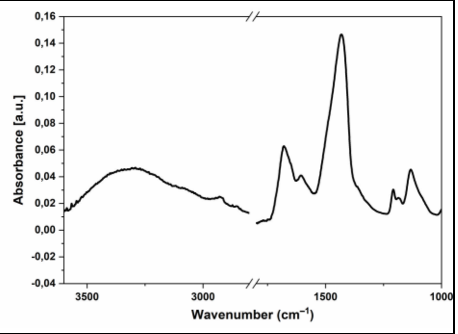
9	HLFNLT	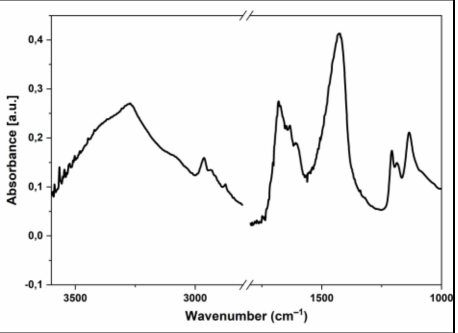
10	HSSNNF	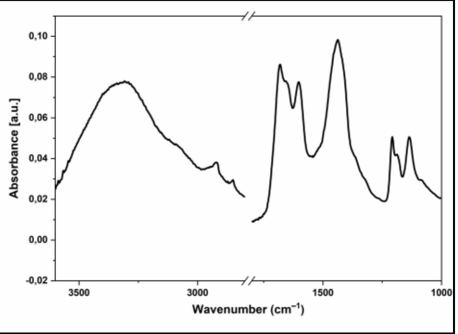
11	MIENIQ	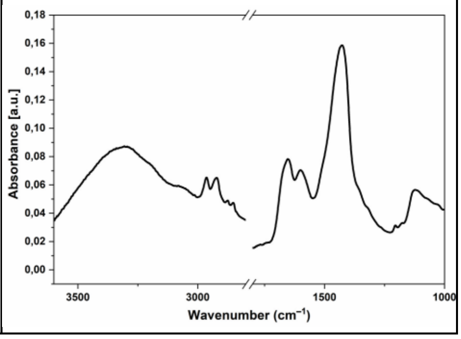

12	MIHFGN	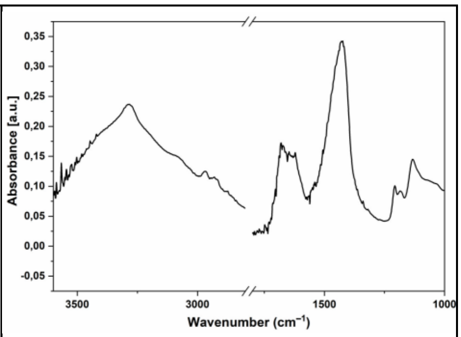
13	MMHFGN	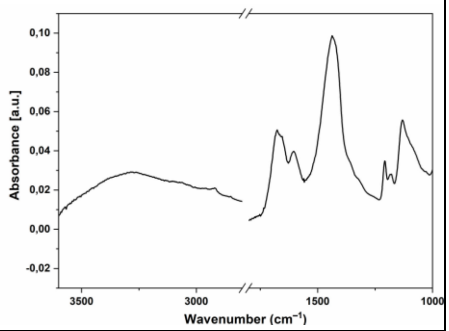
14	NIFNIT	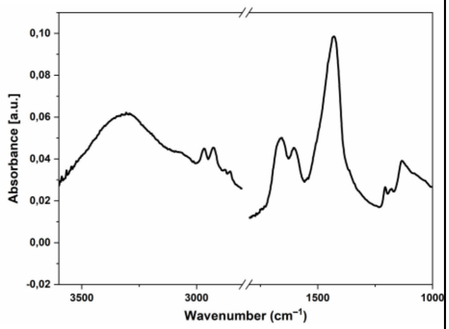
15	NNSGPN	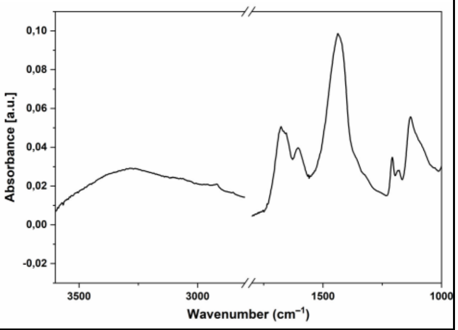
16	NTIFVQ	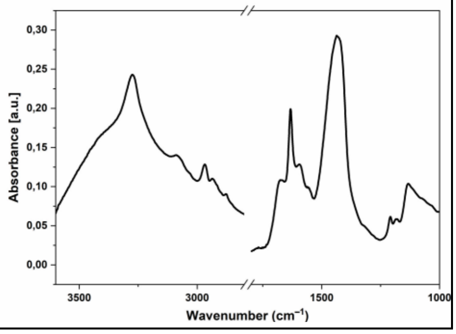

17	QANKHI	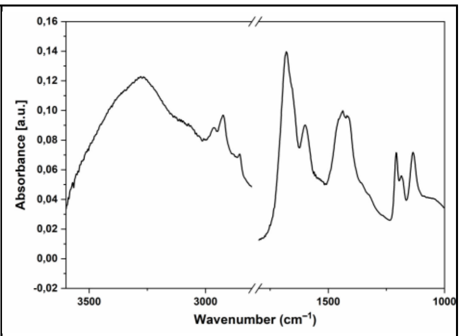
18	QEMRHF	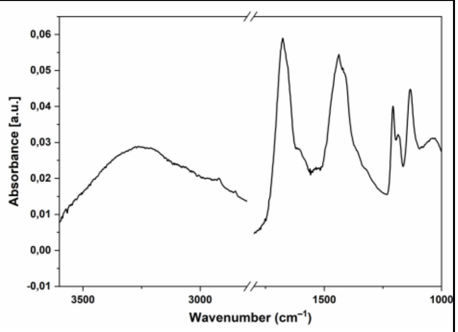
19	SHVIIE	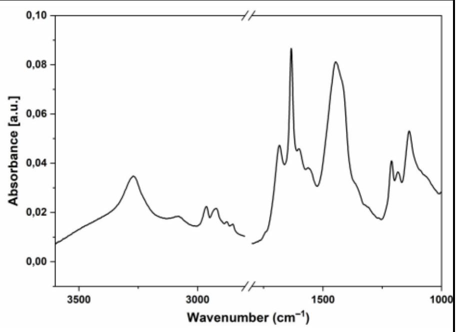
20	STTIIE	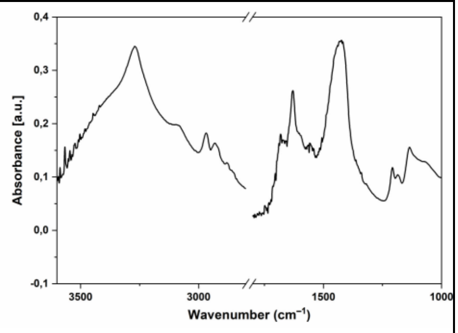
21	STVVIE	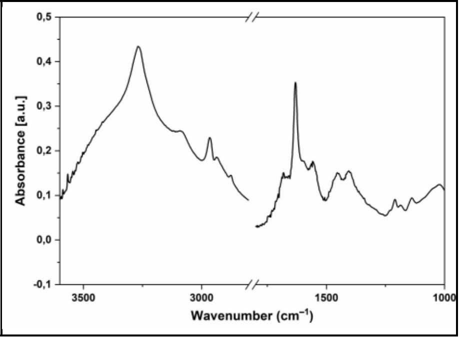

22	SWVIIE	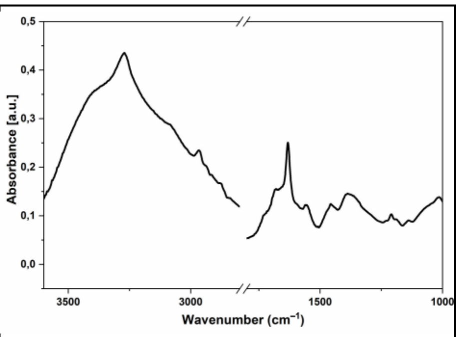
23	WSFYLL	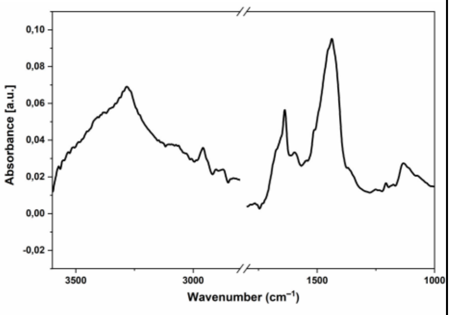
24	YYTEFT	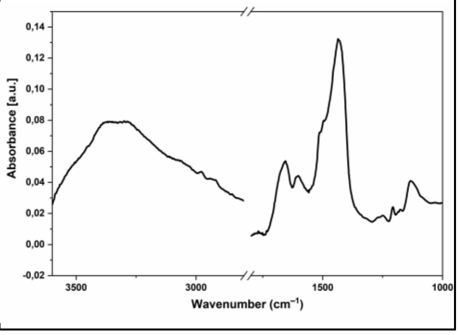

3.1.1.2. Amide spectra with second derivative

Table 9 Amide I spectrum with second derivative

<table>
<tr><th>No.</th><th>Sequence</th><th>ATR-FTIR</th></tr>
<tr><td>1</td><td>ALEEYT</td><td>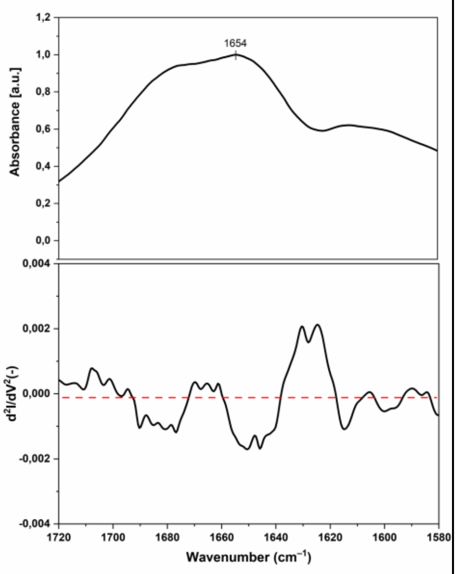
</td></tr>
<tr><td>2</td><td>ASSSNY</td><td>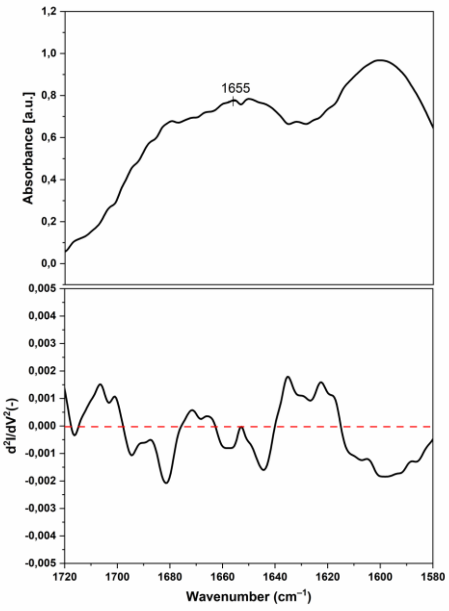
</td></tr>
</table>

3	DETVIV	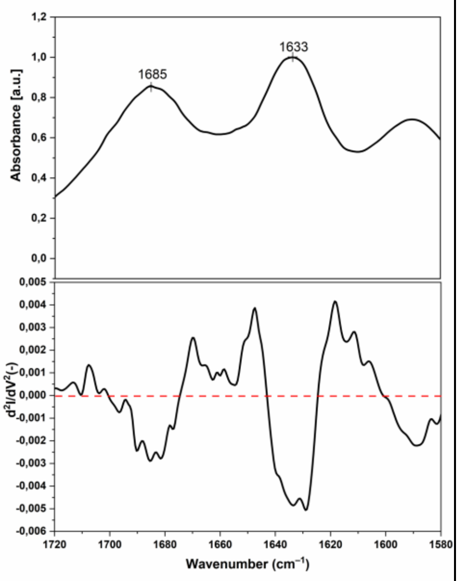
4	ELNIYQ	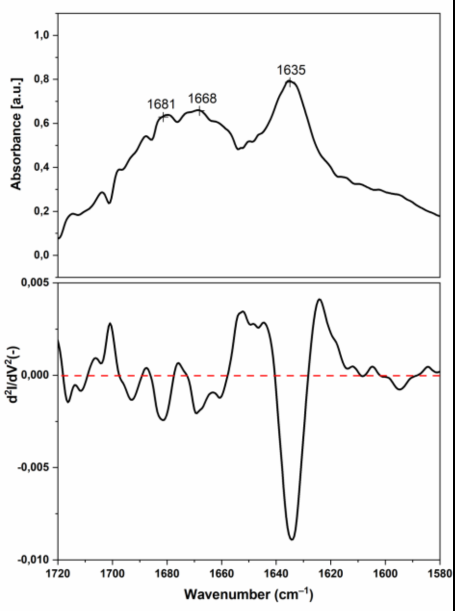

5	FGELFE	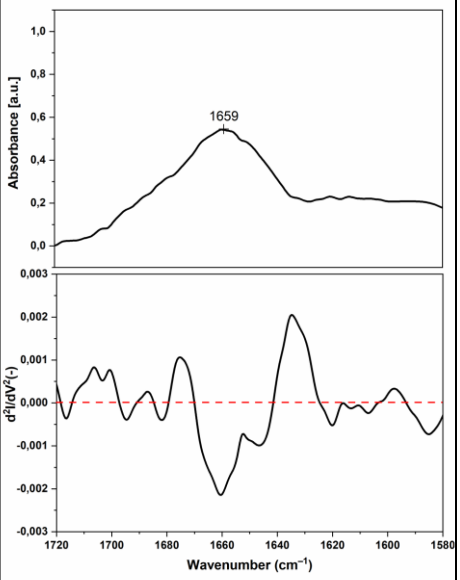
6	FQKQQK	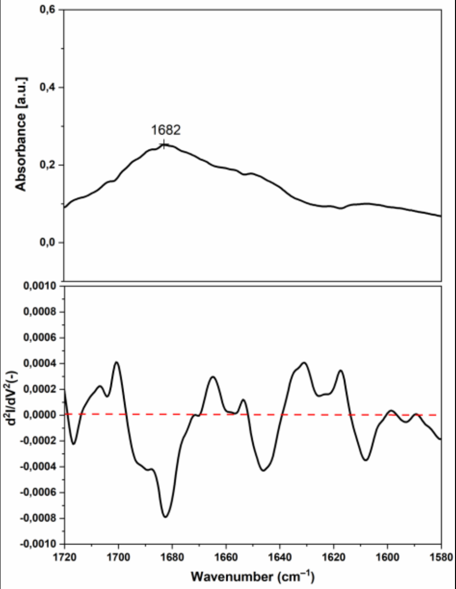

7	FTPTEK	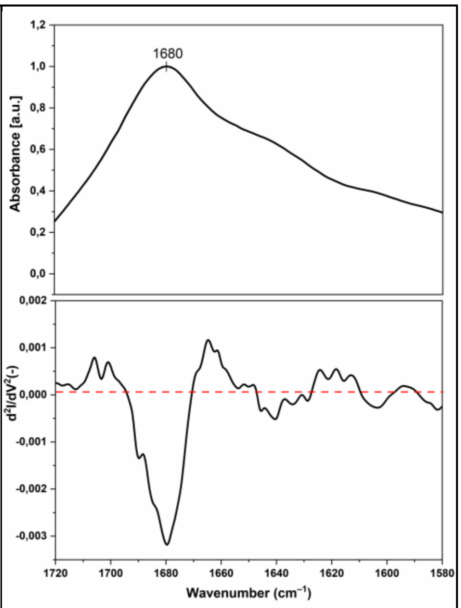
8	HGFNQQ	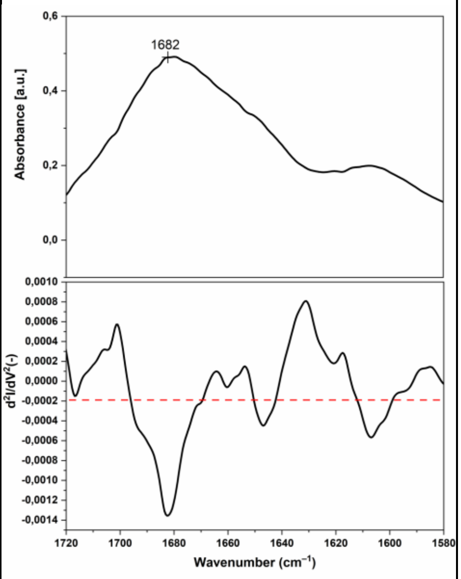

<table>
<tr><td>9</td><td>HLFNLT</td><td>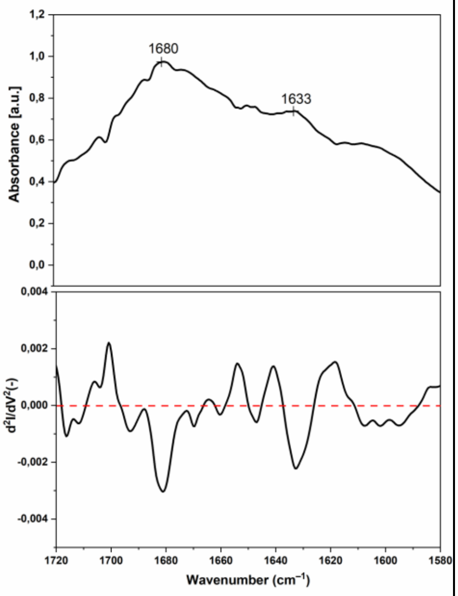</td></tr>
<tr><td>10</td><td>HSSNNF</td><td>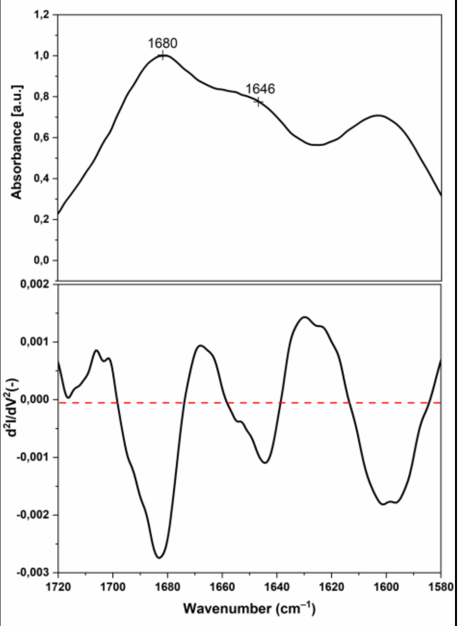</td></tr>
</table>

11	MIENIQ	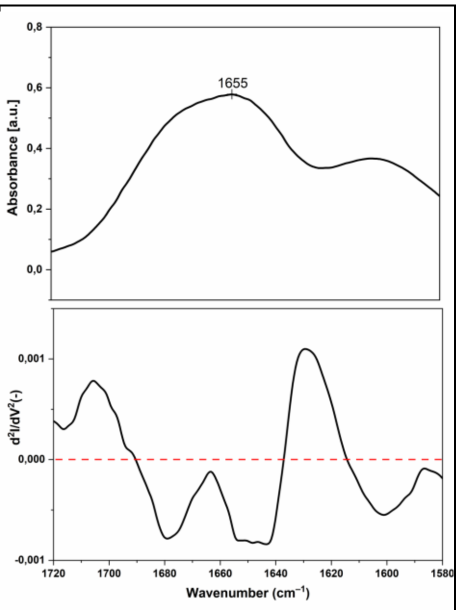
12	MIHFGN	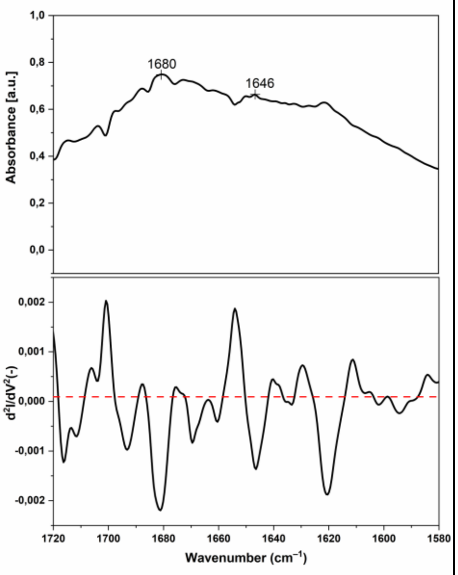

13	MMHFGN	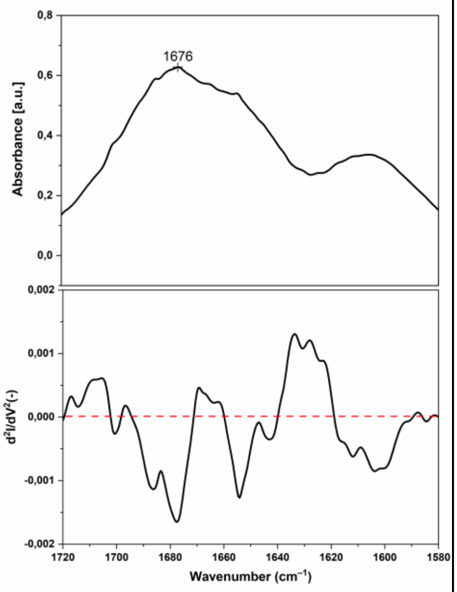
14	NIFNIT	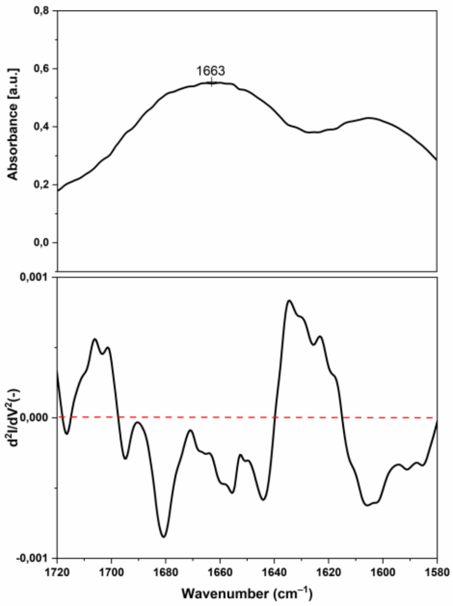

<table>
<tr><td>15</td><td>NNSGPN</td><td>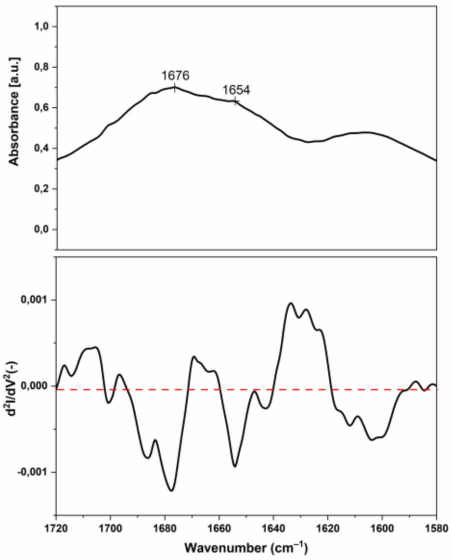
</td></tr>
<tr><td>16</td><td>NTIFVQ</td><td>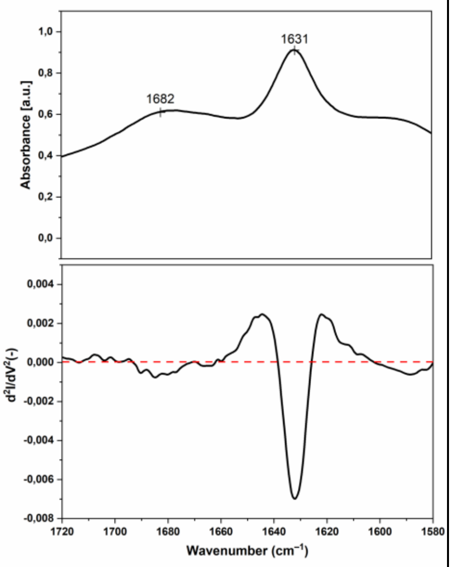
</td></tr>
</table>

17	QANKHI	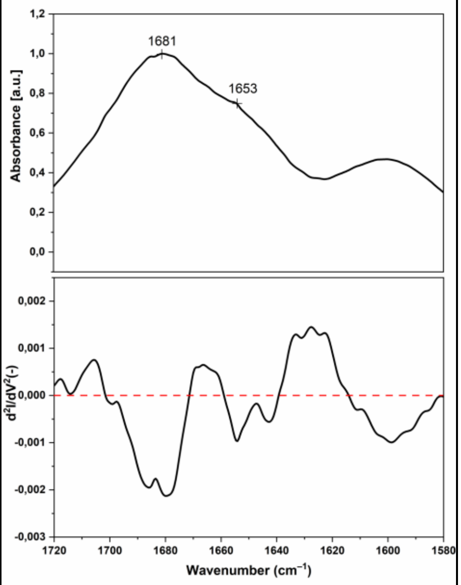
18	QEMRHF	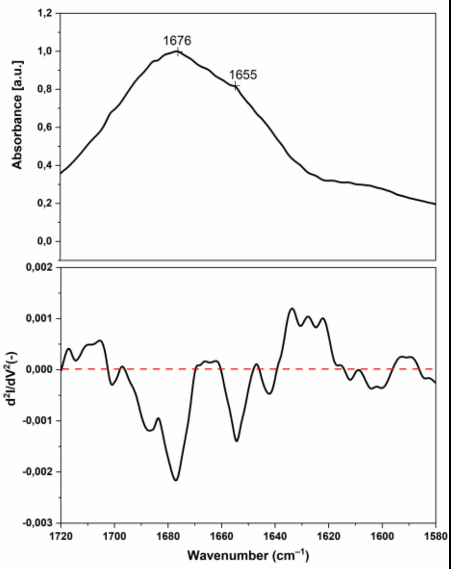

19	SHVIIE	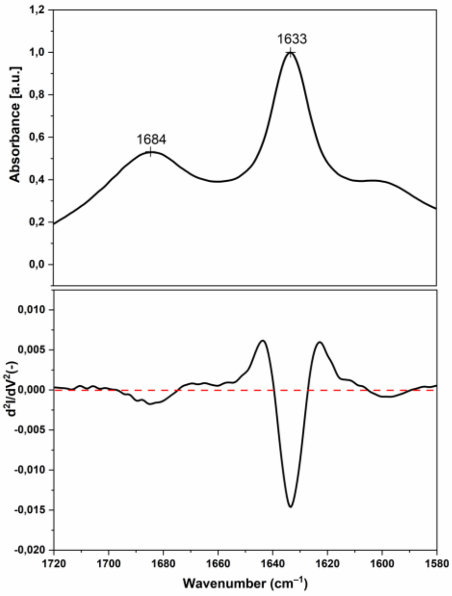
20	STTIIE	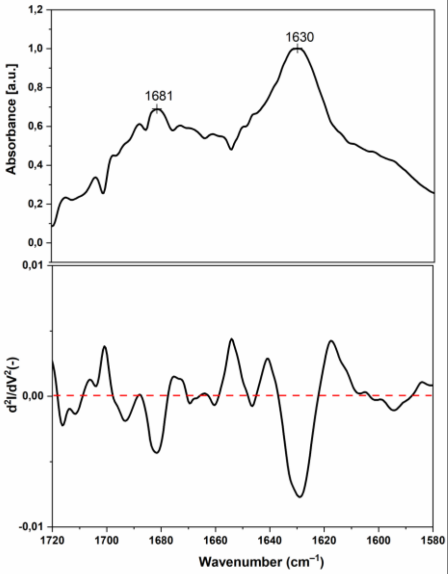

<table>
<tr>
<td>21</td>
<td>STVVIE</td>
<td>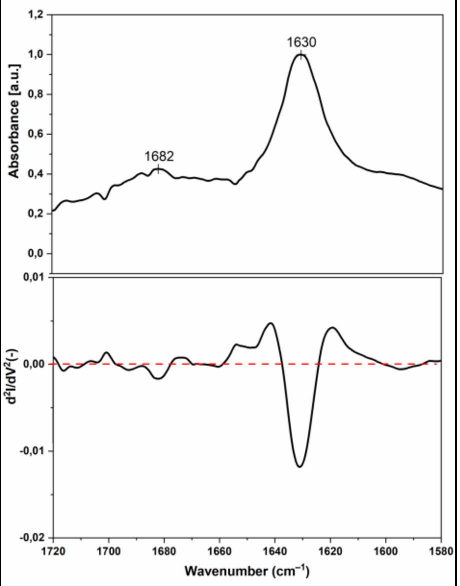
</td>
</tr>
<tr>
<td>22</td>
<td>SWVIIE</td>
<td>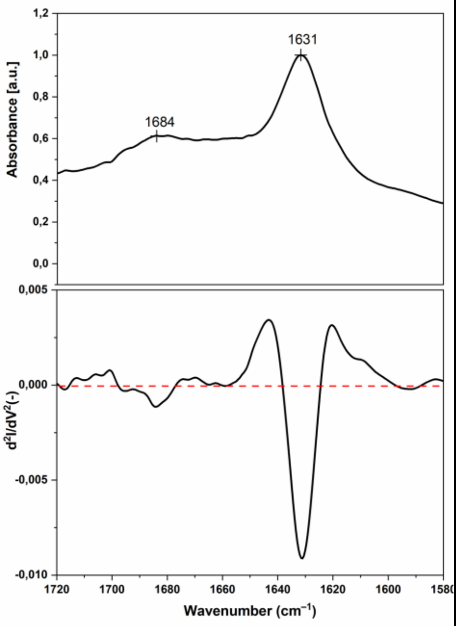
</td>
</tr>
</table>

<table>
<tr><td>23</td><td>WSFYLL</td><td>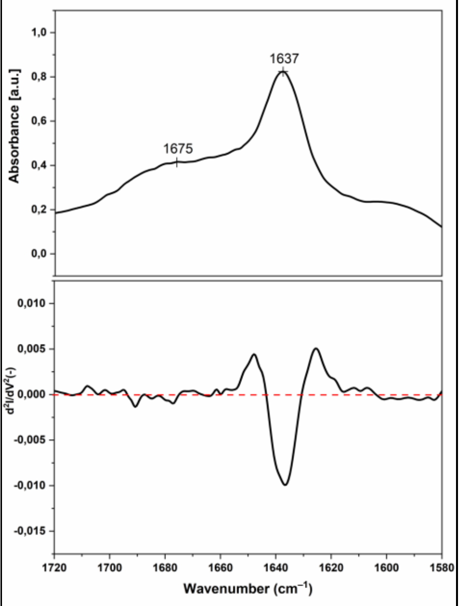
</td></tr>
<tr><td>24</td><td>YYTEFT</td><td>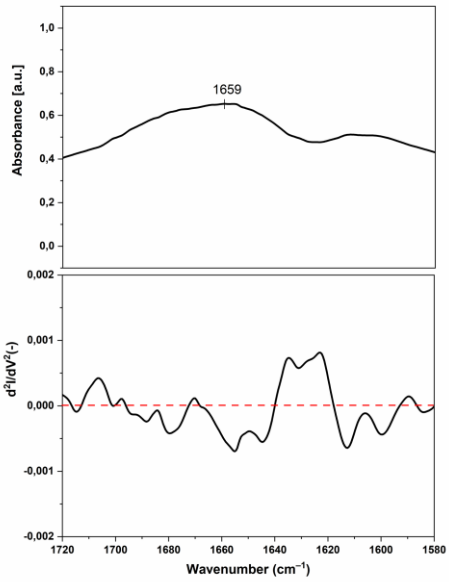
</td></tr>
</table>

3.1.2. Infrared Microscopy using transmission mode (IR microscopy)

3.1.2.1. Normalized spectra in the range of 3600-1000 cm^{-1}

Table 10 All spectra of examined hexapeptides

No.	Sequence	IR microscopy
1	ALEEYT	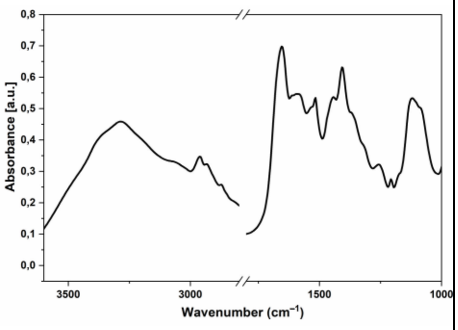
2	ASSSNY	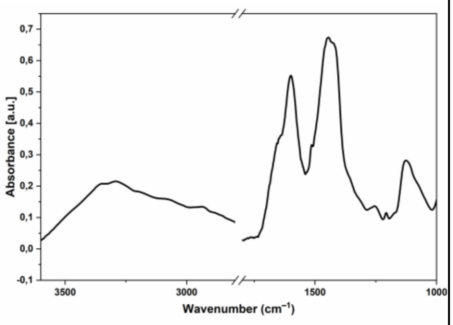
3	DETVIV	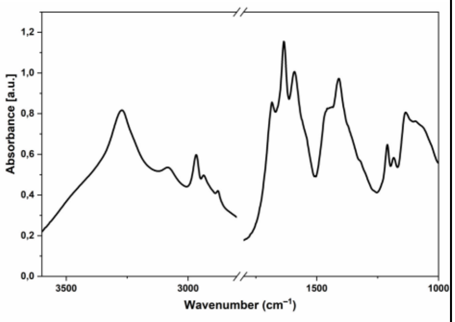
4	ELNIYQ	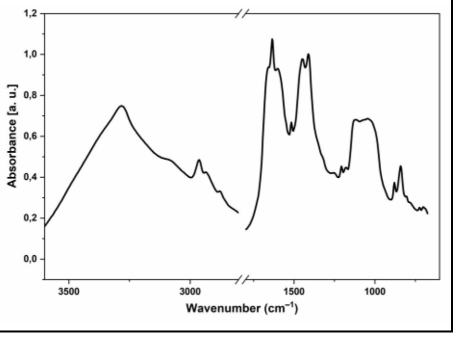

5	FGELFE	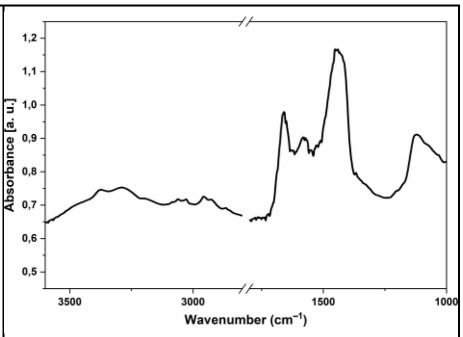
6	FQKQQK	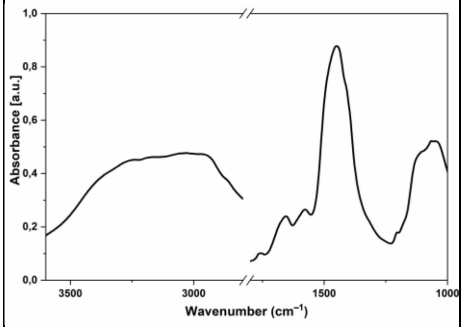
7	FTPTEK	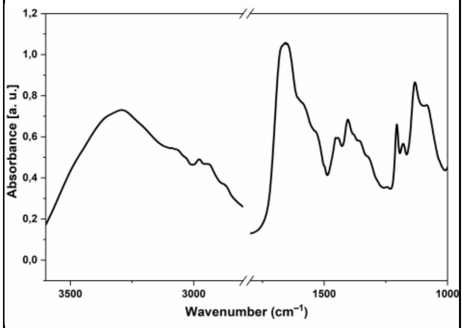
8	HGFNQQ	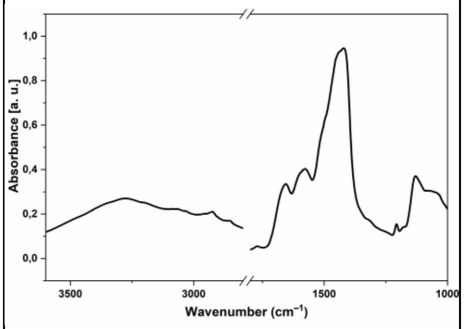
9	HLFNLT	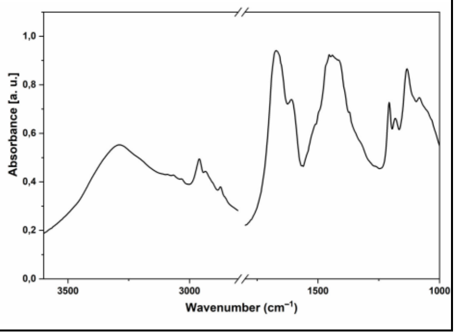

10	HSSNNF	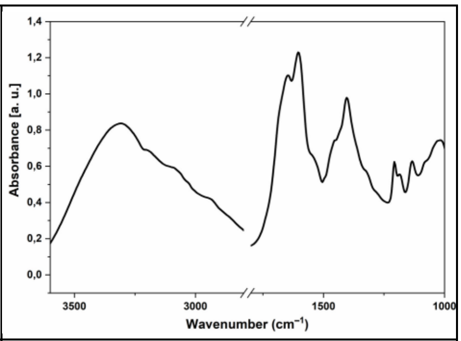
11	MIENIQ	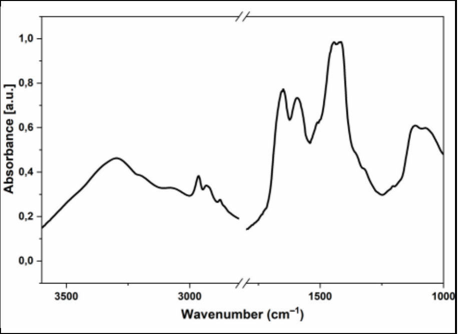
12	MIHFGN	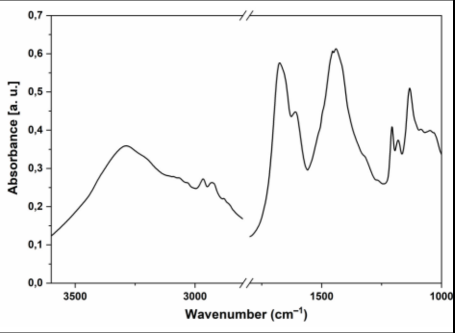
13	MMHFGN	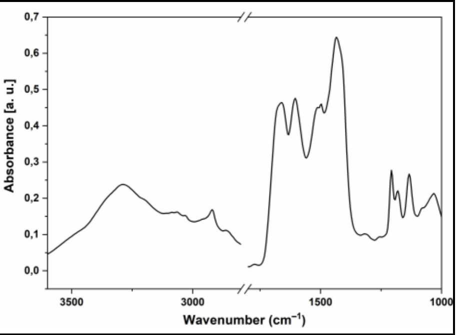
14	NIFNIT	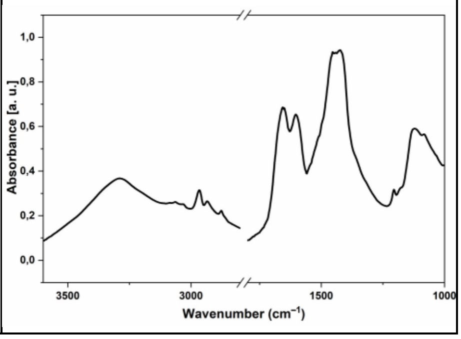

15	NNSGPN	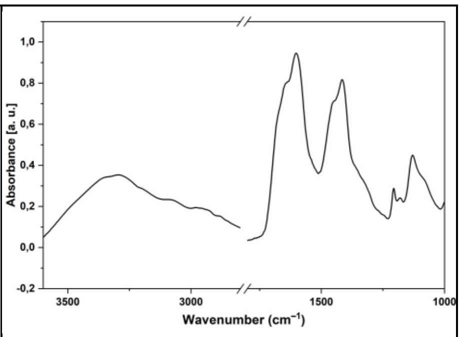
16	NTIFVQ	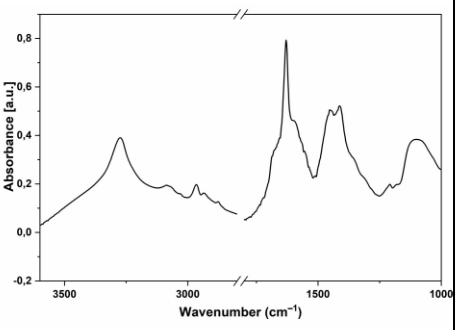
17	QANKHI	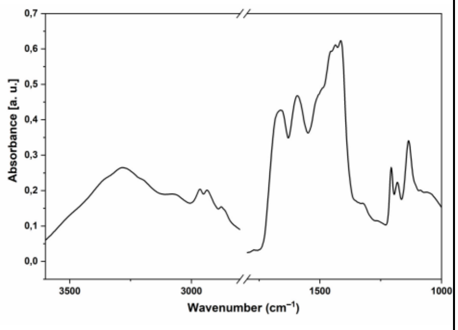
18	QEMRHF	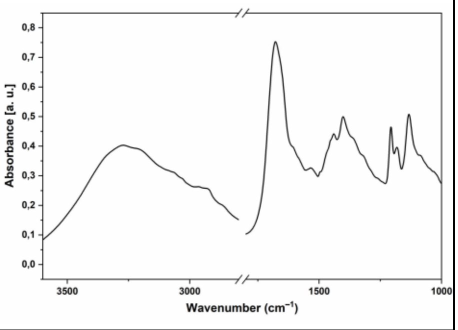
19	SHVIIE	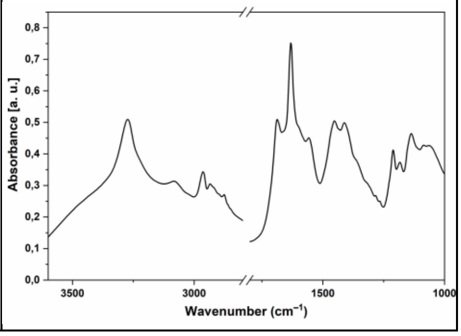

20	STTIIE	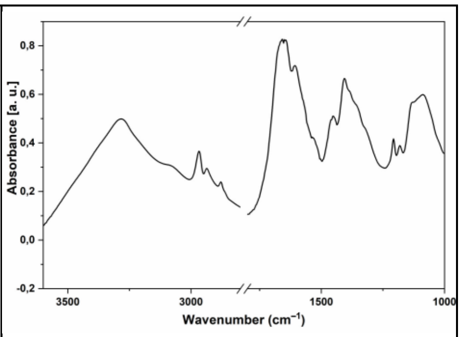
21	STVVIE	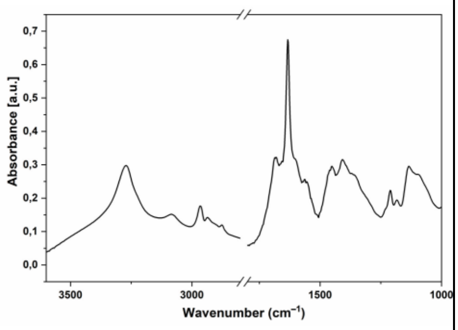
22	SWVIIE	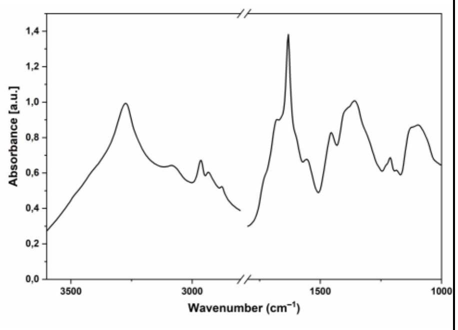
23	WSFYLL	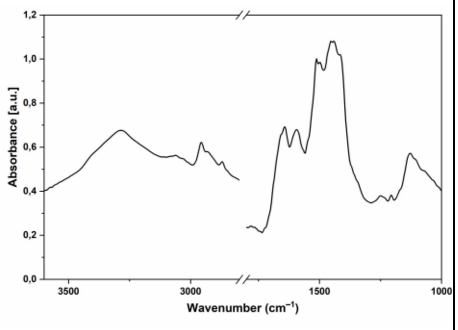
24	YYTEFT	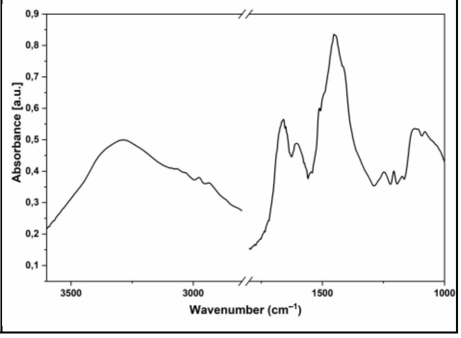

3.1.2.2. Amide spectra with second derivative

Table 11 Amide I spectrum with second derivative

No.	Sequence	IR microscopy
1	ALEEYT	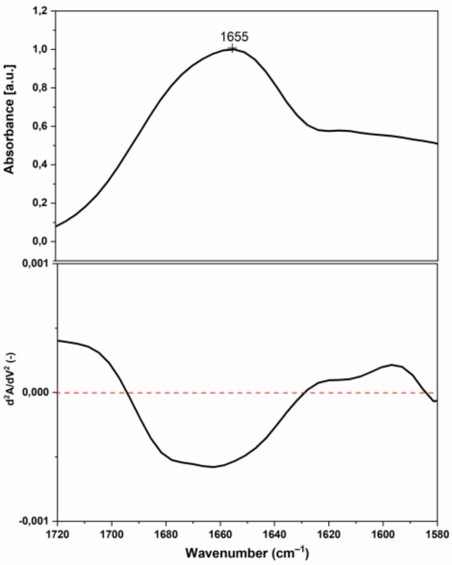
2	ASSSNY	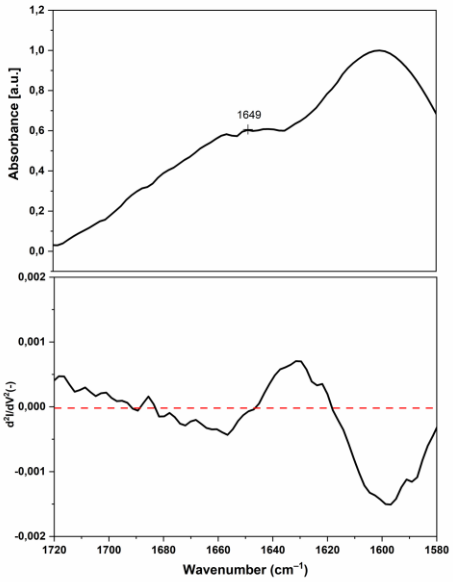

3	DETVIV	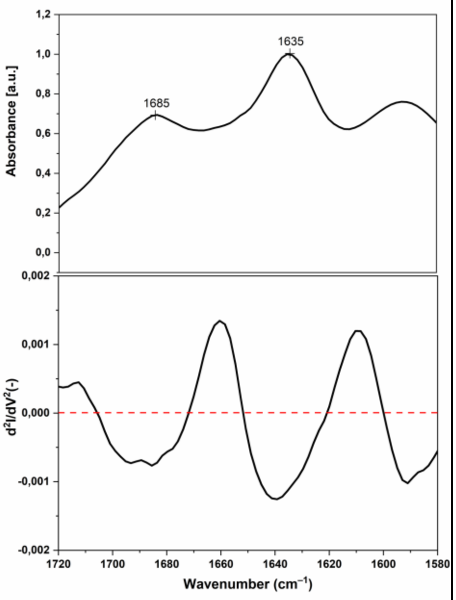
4	ELNIYQ	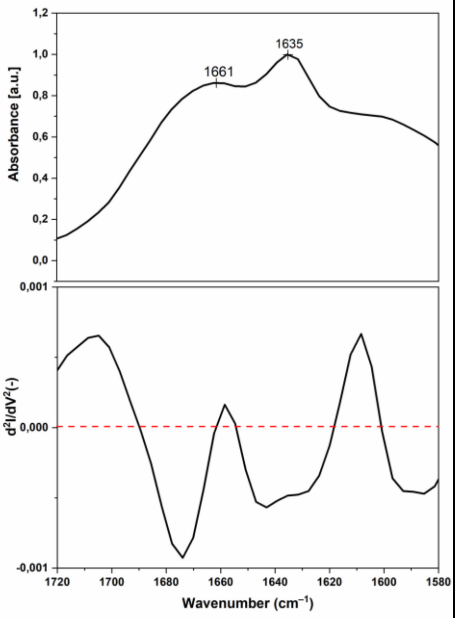

<table>
<tr><td>5</td><td>FGELFE</td><td>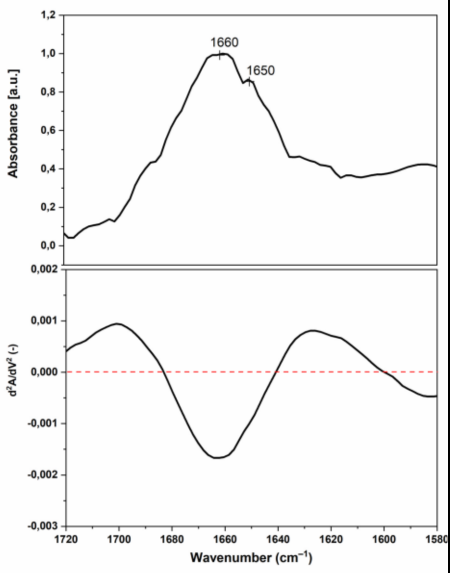
</td></tr>
<tr><td>6</td><td>FQKQQK</td><td>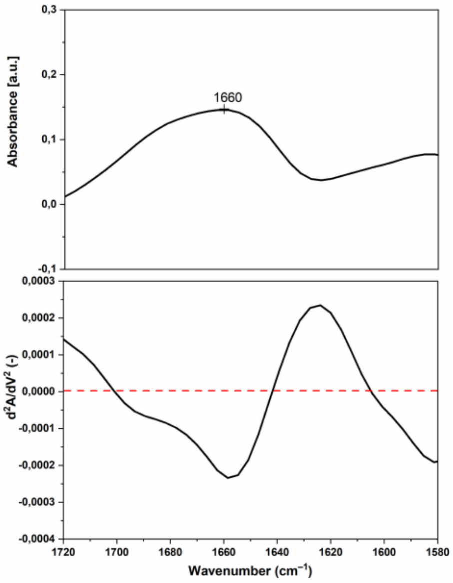
</td></tr>
</table>

7	FTPTEK	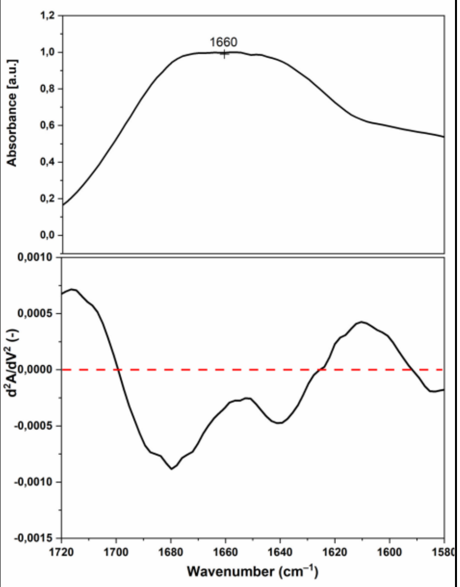
8	HGFNQQ	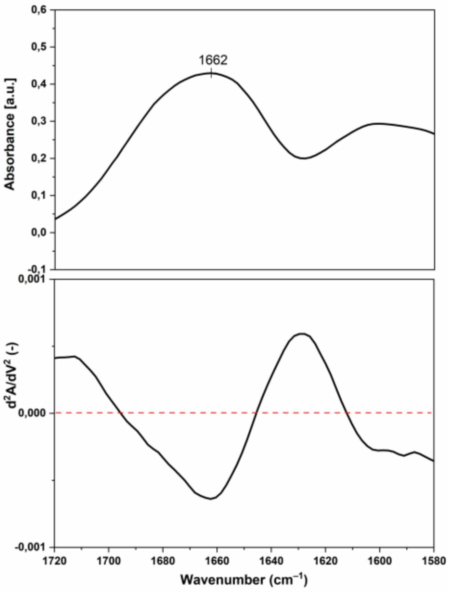

9	HLFNLT	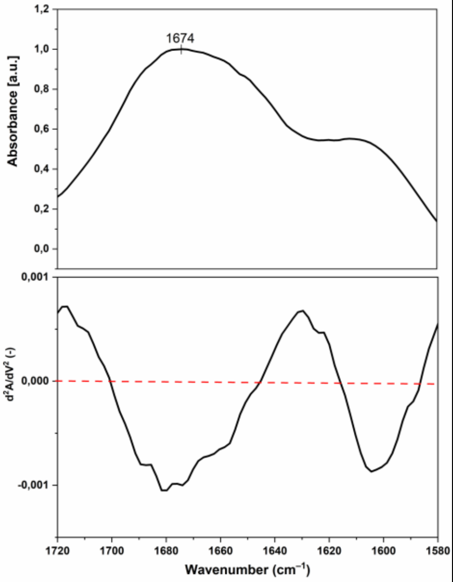
10	HSSNNF	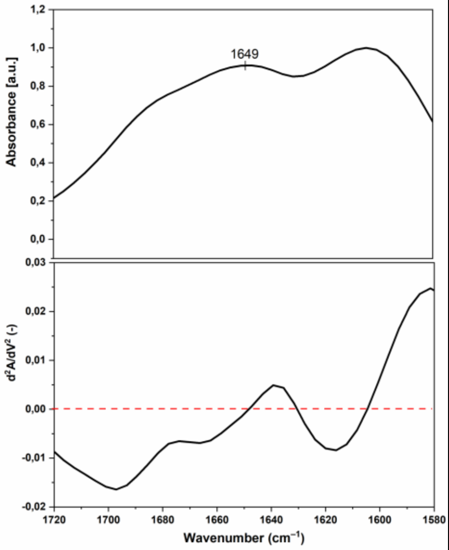

11	MIENIQ	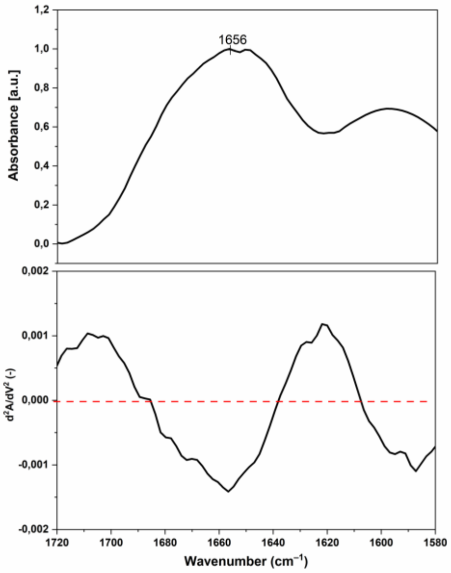
12	MIHFGN	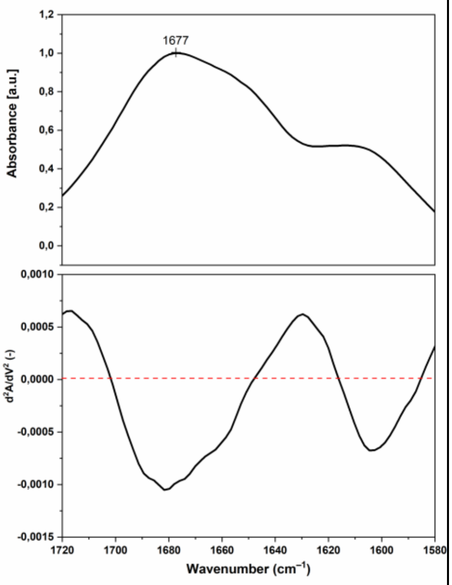

13	MMHFGN	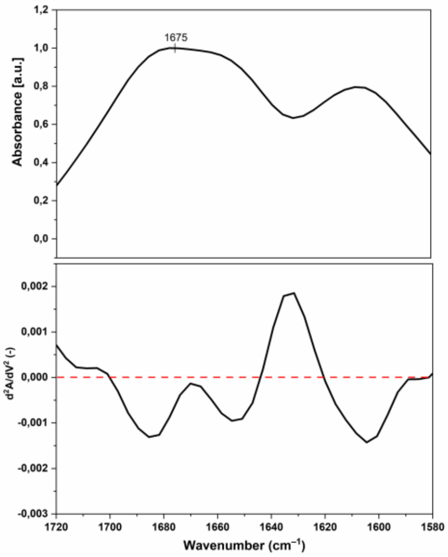
14	NIFNIT	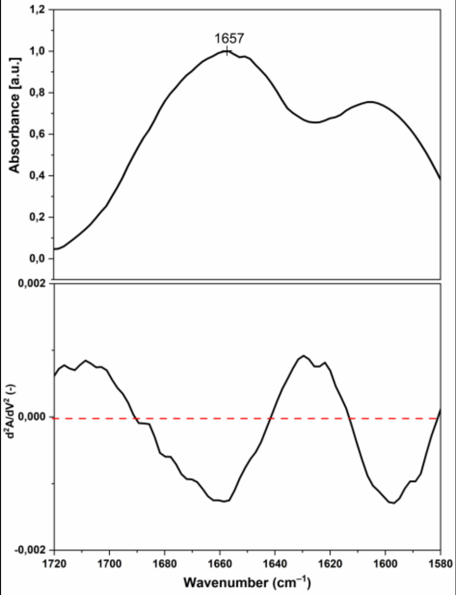

15	NNSGPN	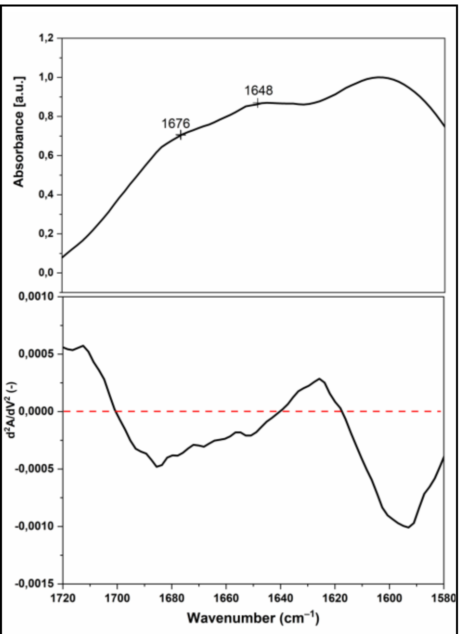
16	NTIFVQ	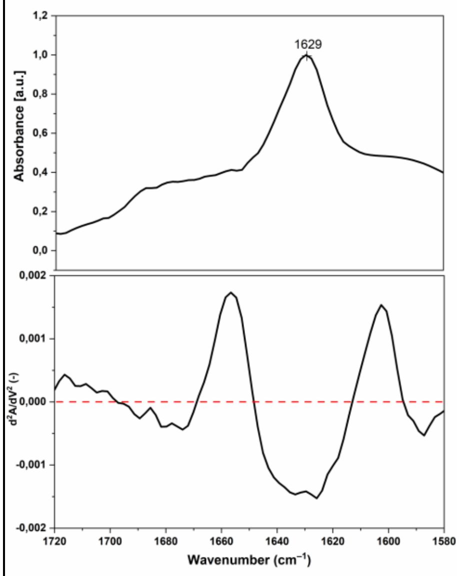

17	QANKHI	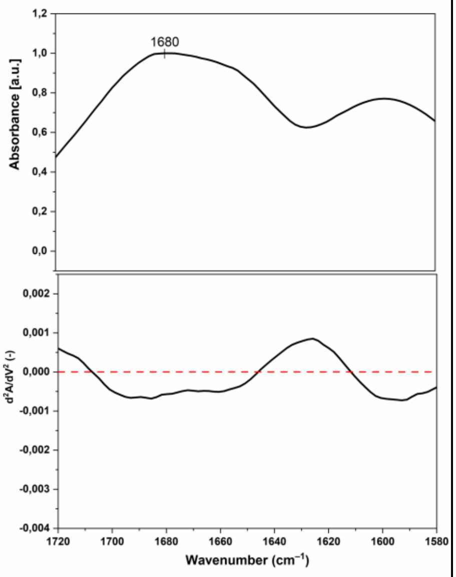
18	QEMRHF	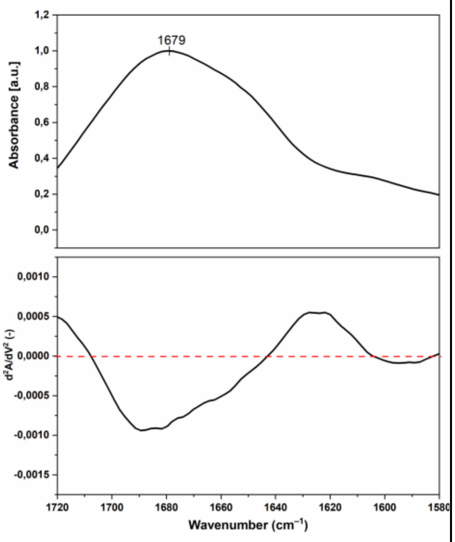

19	SHVIIE	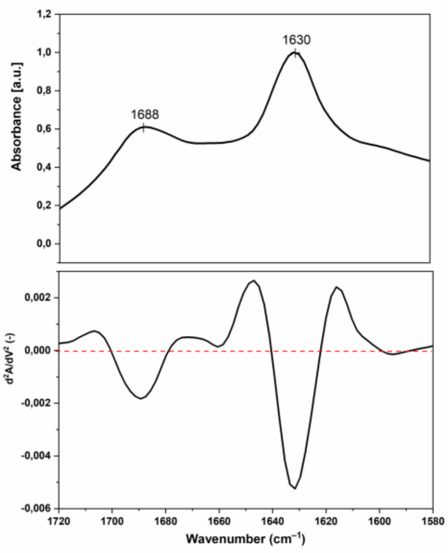
20	STTIIE	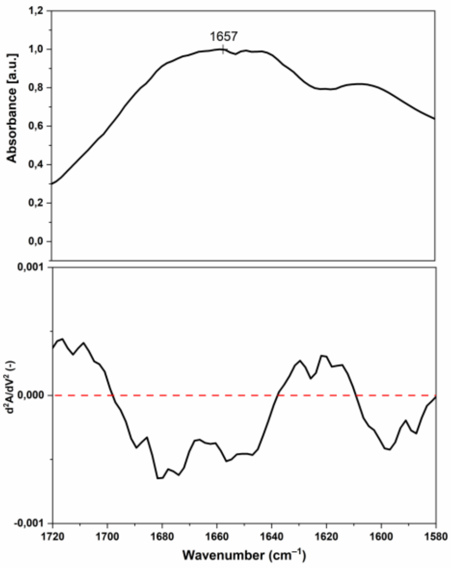

21	STVVIE	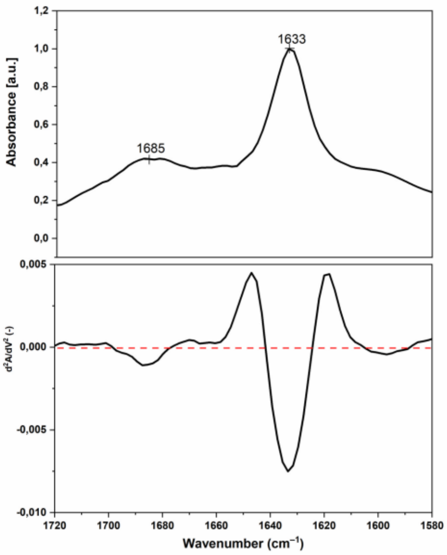
22	SWVIIE	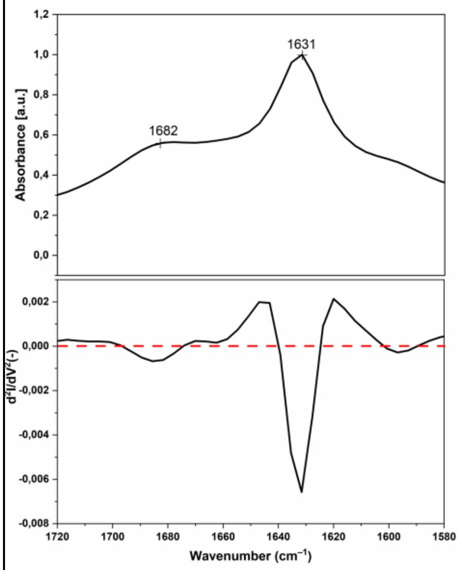

23	WSFYLL	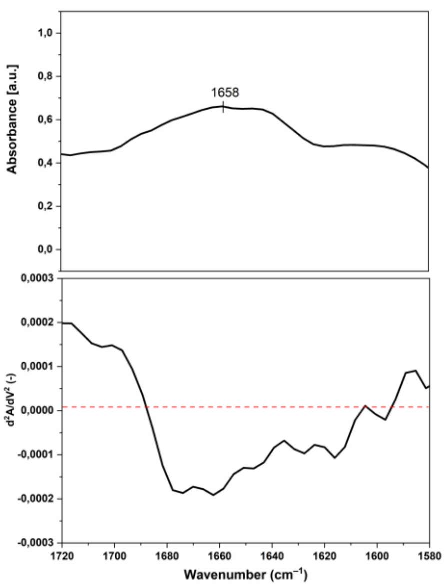
24	YYTEFT	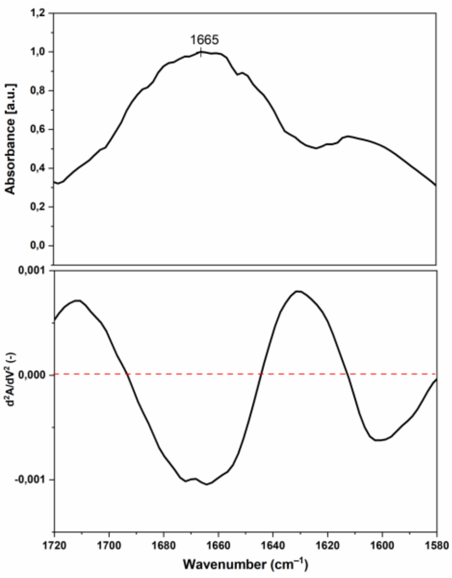

3.2. IR microscopy vs ATR-FTIR

Table 12 Differences between FTIR (transmission mode) and ATR-FTIR spectra of examined hexapeptides

<table>
<tr><th>No.</th><th>Sequence</th><th>IR microscopy vs ATR-FTIR</th></tr>
<tr><td>1</td><td>ALEEYT</td><td>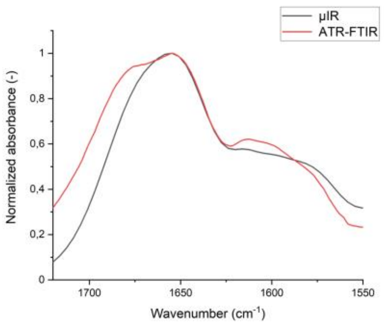
</td></tr>
<tr><td>2</td><td>ASSSNY</td><td>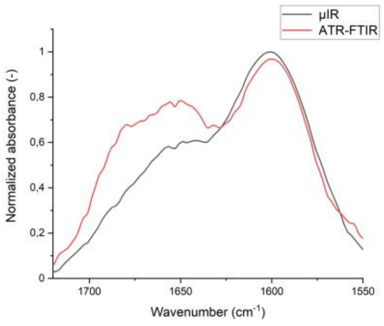
</td></tr>
<tr><td>3</td><td>DETVIV</td><td>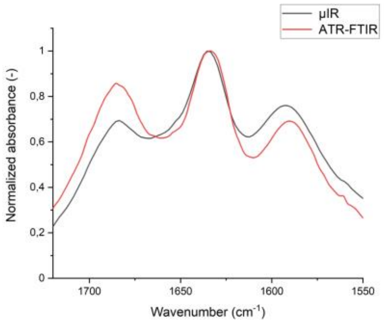
</td></tr>
<tr><td>4</td><td>ELNIYQ</td><td>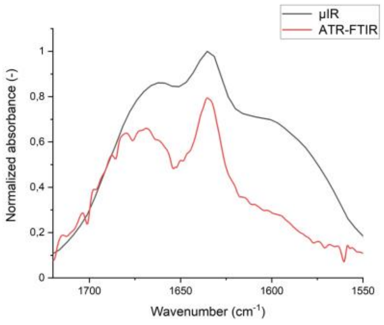
</td></tr>
</table>

5	FGELFE	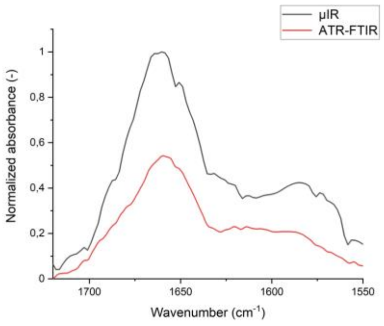
6	FQKQQK	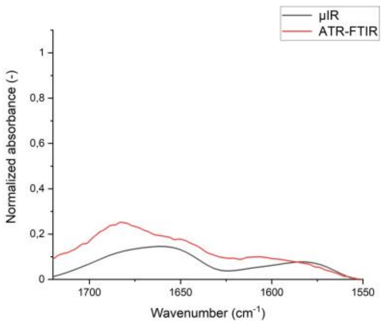
7	FTPTEK	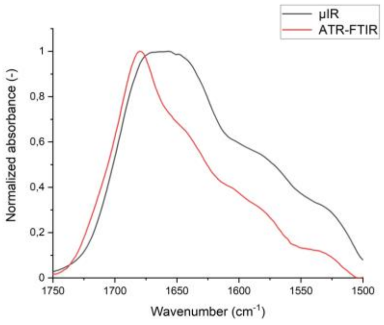
8	HGFNQQ	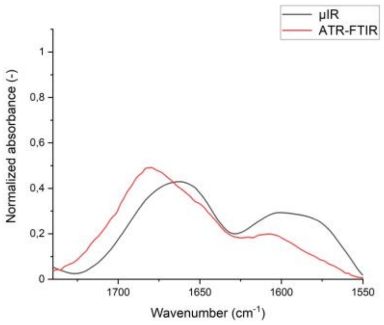
9	HLFNLT	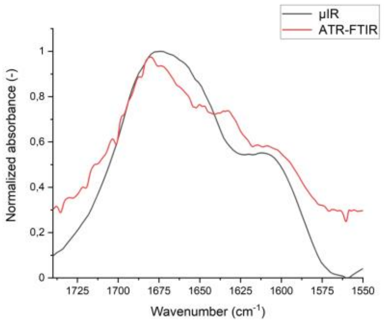

<table>
<tr><td>10</td><td>HSSNNF</td><td>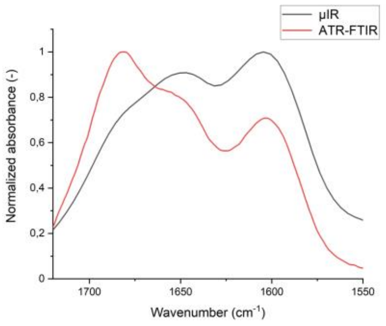
</td></tr>
<tr><td>11</td><td>MIENIQ</td><td>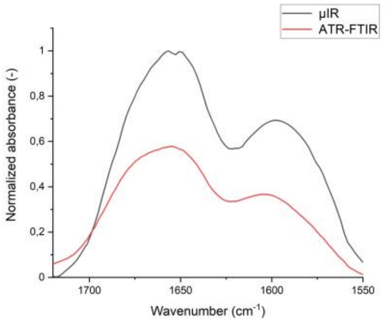
</td></tr>
<tr><td>12</td><td>MIHFGN</td><td>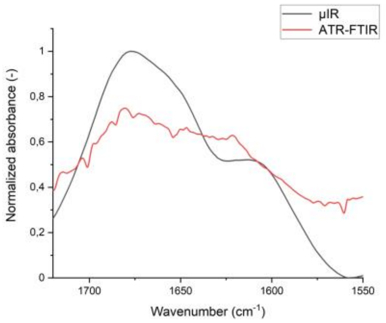
</td></tr>
<tr><td>13</td><td>MMHFGN</td><td>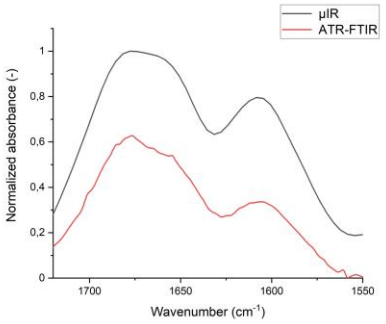
</td></tr>
<tr><td>14</td><td>NIFNIT</td><td>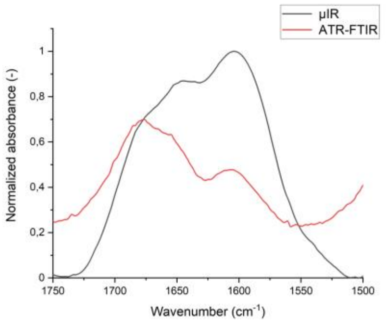
</td></tr>
</table>

<table>
<tr><td>15</td><td>NNSGPN</td><td>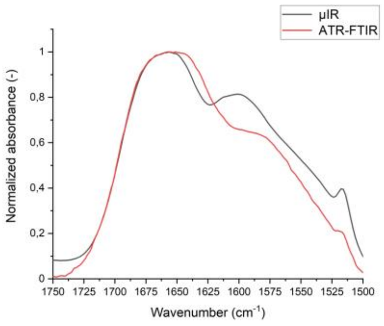
</td></tr>
<tr><td>16</td><td>NTIFVQ</td><td>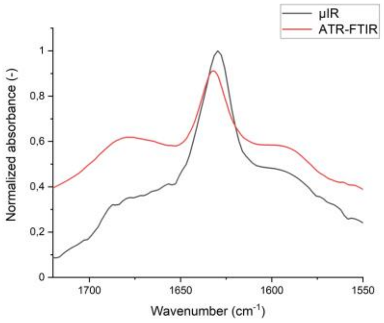
</td></tr>
<tr><td>17</td><td>QANKHI</td><td>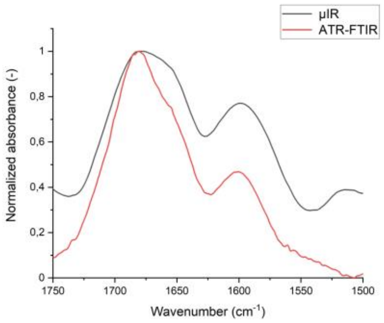
</td></tr>
<tr><td>18</td><td>QEMRHF</td><td>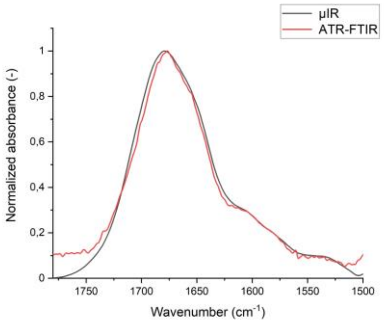
</td></tr>
<tr><td>19</td><td>SHVIIE</td><td>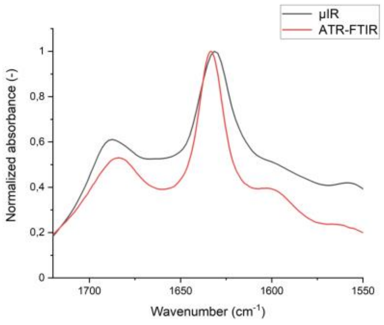
</td></tr>
</table>

20	STTIIE	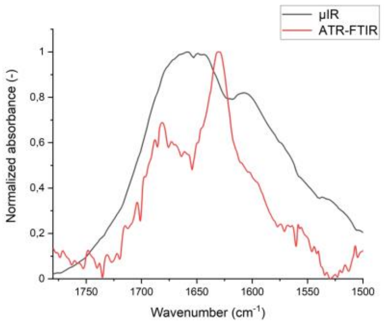
21	STVVIE	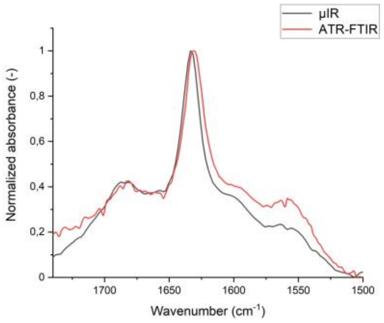
22	SWVIIE	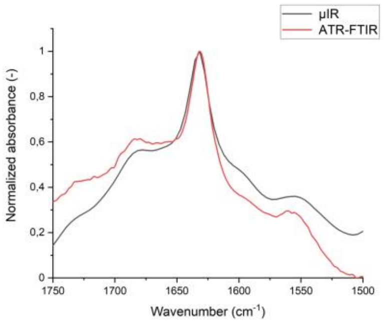
23	WSFYLL	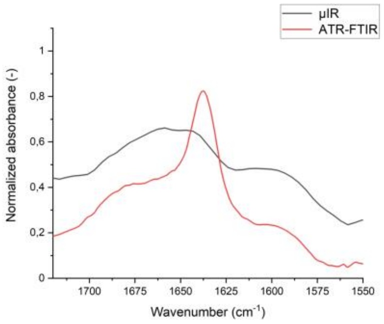
24	YYTEFT	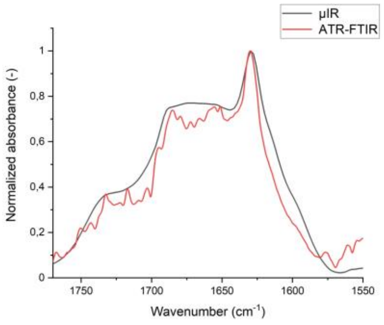

3.3. PCA analysis

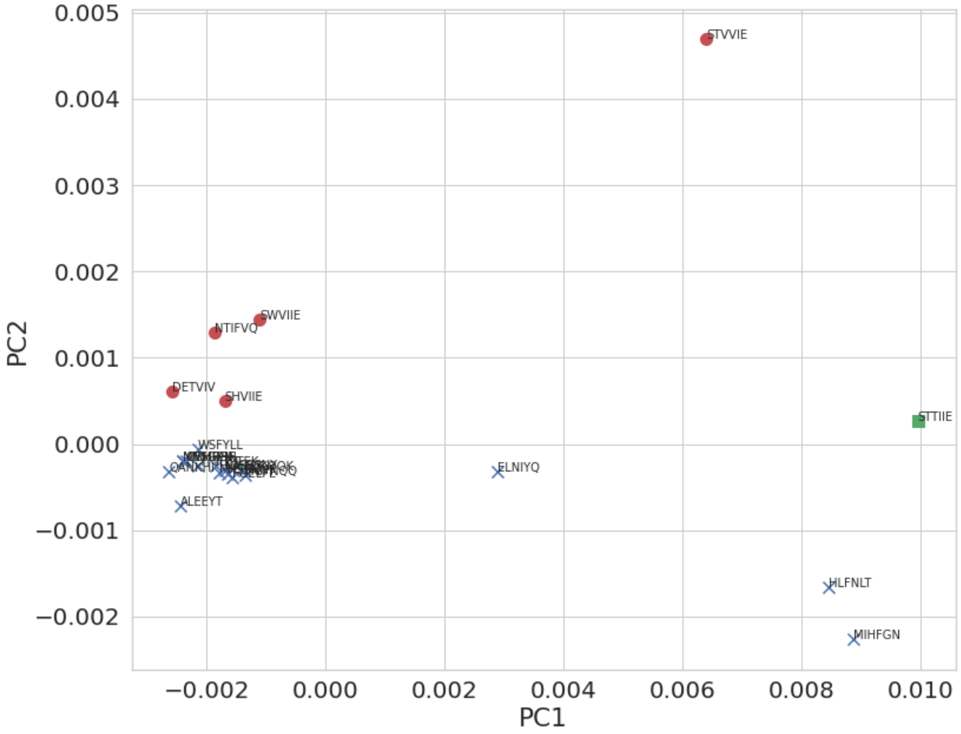

Figure 5 PCA plot for ATR-FTIR spectra of the test set. Red dot assigned to amyloid, blue cross to non-amyloid and green square as ambiguous.

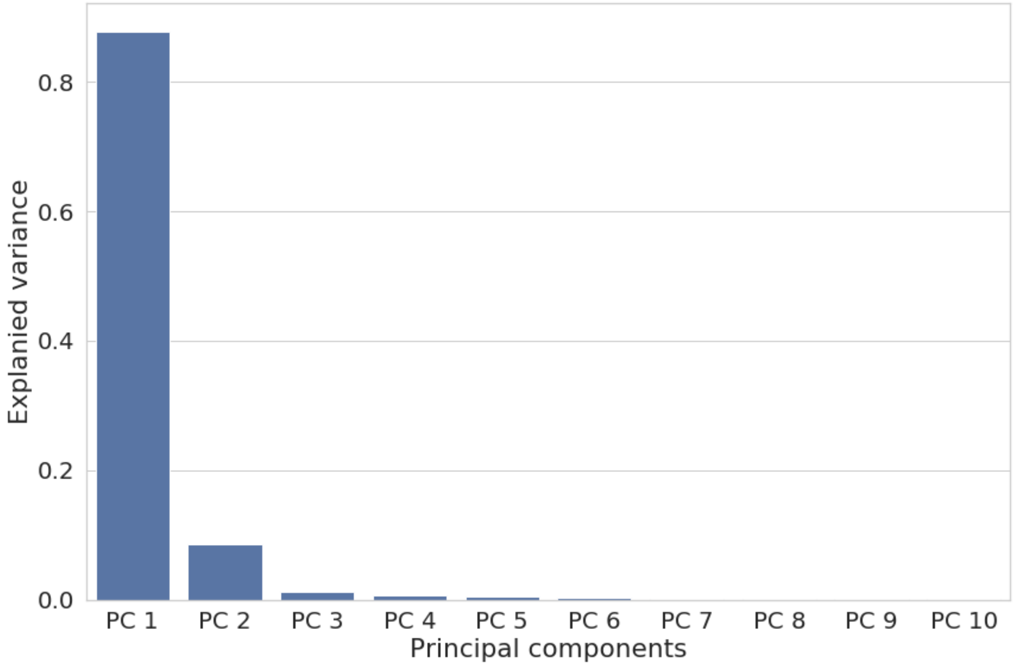

Figure 6 The distribution of principal components for ATR-FTIR spectra in test set.

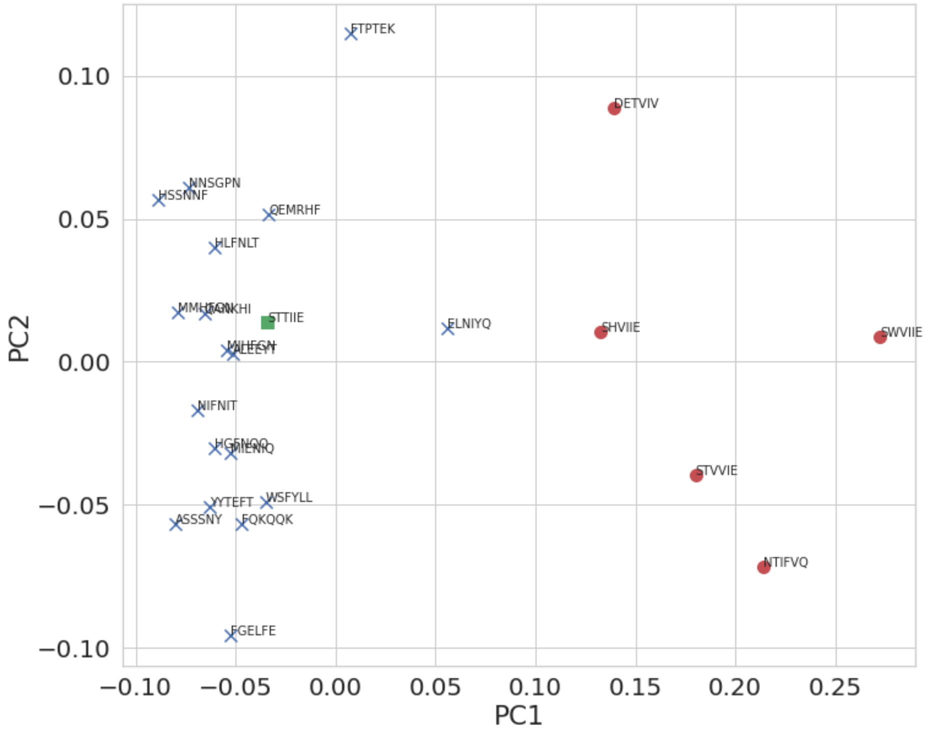

Figure 7 PCA plot for IR microscopy spectra of the test set. Red dot assigned to amyloid, blue cross to non-amyloid and green square as ambiguous.

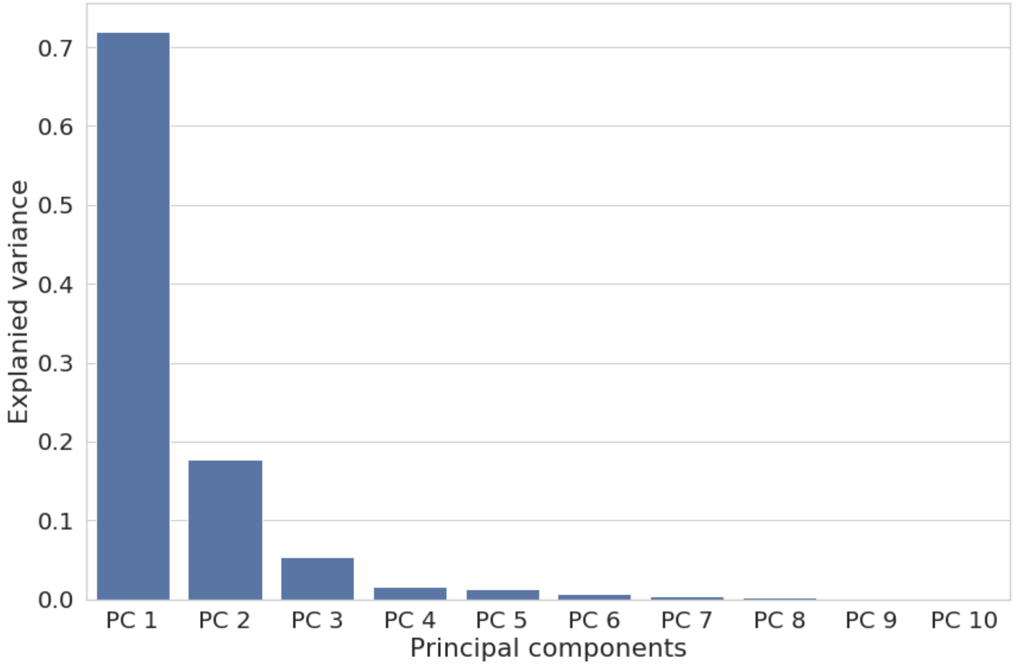

Figure 8 The distribution of principal components for IR microscopy spectra in test set.
