# Supplementary material for: Bioinformatics methods for identification of amyloidogenic peptides show robustness to misannotated training data
Source: Sci Rep. 2021 Apr 26;11:8934. doi: 10.1038/s41598-021-86530-6 (PMC8076271; doi:10.1038/s41598-021-86530-6)
Supplement: Supplementary file 2 — Supplementary Information 2. [file 41598_2021_86530_MOESM2_ESM.pdf]

Supplement 2

Bioinformatic methods for identification of amyloidogenic peptides shows robustness to misannotated training data

Table 1 IR microscopy based classification versus the source database, and potential sources of discrepancies

No	Sequence	DB	IR microscopy	Comments and potential source of discrepancy DB vs IR	AmyloGram
1	ALEEYT	**yes**	**no**	Literature based (EM, TEM), specific results not shown	no (0.07)
2	ASSSNY	**yes**	**no**	Literature based, available EM only	no (0.09)
3	DETVIV	**no**	**yes***	WaltzDB unpublished data (FTIR, EM), EM shows a fibril-like structure, FTIR does not support fibrils	yes (0.67)
4	ELNIYQ	no	no	Agreed, WaltzDB unpublished data (no results shown)	yes (0.82)
5	FGELFE	no	no	Agreed, but peptide not available in the cited literature	yes (0.65)
6	FQKQQK	no	no	Agreed, Waltz DB unpublished data (no results shown)	yes (0.67)
7	FTPTEK	no	no	Agreed, literature based	yes (0.67)
8	HGFNQQ	**yes**	**no**	WaltzDB unpublished data (FTIR does not support fibrils, EM – possible fibrils)	no (0.08)
9	HLFNLT	**yes**	**no**	WaltzDB unpublished data (does not support fibrils, EM – possible fibrils)	no (0.14)
10	HSSNNF	**yes**	**no**	Literature based, available EM only	no (0.09)
11	MIENIQ	**yes**	**no**	Possible DB misprint, peptide not available in the cited literature	no (0.1)
12	MIHFGN	**yes**	**no**	Literature based, available EM only	no (0.14)
13	MMHFGN	**yes**	**no**	Literature based, available EM only	no (0.05)
14	NIFNIT	**yes**	**no**	Literature based, far-UV CD and EM	no (0.12)
15	NNSGPN	**yes**	**no**	WaltzDB unpublished data (does not support fibrils, EM – possible fibrils)	no (0.01)
16	NTIFVQ	**no**	**yes**	WaltzDB unpublished data (no results shown)	yes (0.70)
17	QANKHI	**yes**	**no**	Literature based, available EM only	no (0.04)
18	QEMRHF	**yes**	**no**	Literature based, available EM only	no (0.05)
19	SHVIIE	**no**	**yes**	Possible misprint, peptide not available in the cited literature	yes (0.64)
20	STTIIE	no	no	Agreed, literature based, reported EM conclusion only	yes (0.64)
21	STVVIE	**no**	**yes**	Literature based, mistake in DB annotation (wrong column taken)	yes (0.86)
22	SWVIIE	**no**	**yes**	Literature based, mistake in DB annotation (wrong column taken)	yes (0.62)
23	WSFYLL	no	no	Agreed, available EM only	yes (0.77)
24	YYTEFT	no	no	Agreed, available EM only	yes (0.72)

Table 2 Classification results for the *reference set* with amyloidogenicity scores by AmyloGram and PATH, FoldAmyloid average values for peptide/protein and PASTA 2.0 best energy. Data compiled with IR microscopy results.

No	Sequence	IR microscopy	**AmyloGram** amyloid score **[0; 1]** threshold: **0.5**	**FoldAmyloid** amyloid score **[15; 30]** threshold: **21.4**	**PASTA 2.0** amyloid score **[-∞; +∞]** threshold: **-5**	**PATH** **LR** amyloid score **[0; 1]** threshold: **0.5**	**PATH** **RF** amyloid score **[0; 1]** threshold: **0.5**
1	FNPQGG	no	no (0.0)	no (19.15)	no (3.21)	no (0.003)	no (0.049)
2	FTFIQF	yes	yes (0.81)	yes (24.37)	no (-3.97)	yes (0.629)	yes (0.607)
3	ISFLIF	yes	yes (0.91)	yes (24.9)	yes (-5.81)	yes (0.912)	yes (0.660)
4	KPAESD	no	no (0.0)	no (18.1)	no (3.15)	no (0.005)	no (0.033)
5	LVFYQQ	yes	yes (0.89)	yes (23.81)	no (-4.08)	yes (0.641)	yes (0.542)
6	NPQGGY	no	no (0.0)	no (18.93)	no (1.99)	no (0.020)	no (0.028)
7	SFLIFL	yes*	yes (0.92)	yes (25.09)	yes (-5.26)	yes (0.929)	yes (0.656)
8	TKPAES	no	no (0.0)	no (18.39)	no (2.82)	no (0.013)	no (0.026)
9	YLLYYT	yes	yes (0.91)	yes (24.92)	no (-2.90)	yes (0.540)	yes (0.535)
10	YTVIIE	yes	yes (0.92)	yes (23.33)	yes (-6.46)	no (0.407)	yes (0.581)

Table 3 Classification results for the *test set* with amyloidogenicity scores by AmyloGram, FoldAmyloid average values for peptide/protein and PASTA 2.0 best energy. Data compiled with IR microscopy results.

No	Sequence	IR microscopy	**AmyloGram** amyloid score **[0; 1]** threshold: **0.5**	**FoldAmyloid** amyloid score **[15; 30]** threshold: **21.4**	**PASTA 2.0** amyloid score **[-∞; +∞]** threshold: **-5**	**PATH** **LR** amyloid score **[0; 1]** threshold: **0.5**	**PATH** **RF** amyloid score **[0; 1]** threshold: **0.5**
1	ALEEYT	no	no (0.07)	no (20.77)	no (0.24)	no (0.058)	no (0.049)
2	ASSSNY	no	no (0.09)	no (19.47)	no (0.65)	no (0.063)	no (0.087)
3	DETVIV	yes*	yes (0.67)	no (21.46)	yes (-6.06)	no (0.413)	yes (0.502)
4	ELNIYQ	no	yes (0.82)	yes (22.28)	no (-3.14)	no (0.140)	no (0.221)
5	FGELFE	no	yes (0.65)	no (21.89)	no (0.22)	no (0.060)	no (0.091)
6	FQKQQK	no	yes (0.67)	no (19.74)	no (0.69)	no (0.033)	no (0.092)
7	FTPTEK	no	yes (0.67)	no (19.62)	no (1.43)	no (0.012)	no (0.050)
8	HGFNQQ	no	no (0.08)	no (20.69)	no (0.55)	no (0.079)	no (0.076)
9	HLFNLT	no	no (0.14)	yes (23.12)	no (-2.46)	no (0.407)	yes (0.500)
10	HSSNNF	no	no (0.09)	no (19.94)	no (-0.18)	no (0.045)	no (0.063)
11	MIENIQ	no	no (0.1)	no (21.57)	no (-1.48)	no (0.125)	no (0.183)
12	MIHFGN	no	no (0.14)	no (22.72)	no (-2.96)	no (0.358)	no (0.283)
13	MMHFGN	no	no (0.05)	no (22.58)	no (-1.36)	no (0.167)	no (0.184)
14	NIFNIT	no	no (0.12)	yes (22.82)	no (-4.89)	no (0.400)	yes (0.542)
15	NNSGPN	no	no (0.01)	no (17.97)	no (2.04)	no (0.017)	no (0.061)
16	NTIFVQ	yes	yes (0.70)	yes (22.96)	no (-4.95)	yes (0.549)	yes (0.605)
17	QANKHI	no	no (0.04)	no (20.12)	no (-0.53)	no (0.047)	no (0.081)
18	QEMRHF	no	no (0.05)	no (21.9)	no (-1.13)	no (0.124)	no (0.157)
19	SHVIIE	yes	yes (0.64)	yes (22.63)	yes (-5.87)	no (0.463)	no (0.489)
20	STTIIE	no	yes (0.64)	no (21.47)	no (-4.11)	no (0.279)	no (0.324)
21	STVVIE	yes	yes (0.86)	yes (21.95)	yes (-6.06)	no (0.468)	yes (0.524)
22	SWVIIE	yes	yes (0.62)	yes (23.74)	yes (-6.85)	no (0.476)	yes (0.610)
23	WSFYLL	no	yes (0.77)	yes (25.08)	no (-3.07)	yes (0.710)	yes (0.624)
24	YYTEFT	no	yes (0.72)	no (22.34)	no (-2.35)	no (0.198)	no (0.286)
